# Aryl Hydrocarbon Receptor Signaling Synergizes with TLR/NF-κB-Signaling for Induction of IL-22 Through Canonical and Non-Canonical AhR Pathways

**DOI:** 10.3389/ftox.2021.787360

**Published:** 2022-02-03

**Authors:** Yasuhiro Ishihara, Sarah Y. Kado, Keith J. Bein, Yi He, Arshia A. Pouraryan, Angelika Urban, Thomas Haarmann-Stemmann, Colleen Sweeney, Christoph F. A. Vogel

**Affiliations:** ^1^ Center for Health and the Environment, University of California, Davis, Davis, CA, United States; ^2^ Graduate School of Integrated Arts and Sciences, Hiroshima University, Hiroshima, Japan; ^3^ Leibniz Research Institute for Environmental Medicine, Düsseldorf, Germany; ^4^ Department of Biochemistry and Molecular Medicine, School of Medicine, University of California, Davis, Davis, CA, United States; ^5^ Department of Environmental Toxicology, University of California, Davis, Davis, CA, United States

**Keywords:** AhR, toll-like receptor, IL-22, inflammation, macrophages

## Abstract

Interleukin 22 (IL-22) is critically involved in gut immunity and host defense and primarily produced by activated T cells. In different circumstances IL-22 may contribute to pathological conditions or act as a cancer promoting cytokine secreted by infiltrating immune cells. Here we show that bone marrow-derived macrophages (BMM) express and produce IL-22 after activation of the aryl hydrocarbon receptor (AhR) when cells are activated through the Toll-like receptor (TLR) family. The additional activation of AhR triggered a significant induction of IL-22 in TLR-activated BMM. Deletion and mutation constructs of the IL-22 promoter revealed that a consensus DRE and RelBAhRE binding element are necessary to mediate the synergistic effects of AhR and TLR ligands. Inhibitor studies and analysis of BMM derived from knockout mice confirmed that the synergistic induction of IL-22 by AhR and TLR ligands depend on the expression of AhR and Nuclear Factor-kappa B (NF-κB) member RelB. The exposure to particulate matter (PM) collected from traffic related air pollution (TRAP) and wildfires activated AhR as well as NF-κB signaling and significantly induced the expression of IL-22. In summary this study shows that simultaneous activation of the AhR and NF-κB signaling pathways leads to synergistic and prolonged induction of IL-22 by integrating signals of the canonical and non-canonical AhR pathway.

## Introduction

The aryl hydrocarbon receptor (AhR) plays an important role in regulating immune responses (Stockinger et al., 2014; Gutiérrez-Vázquez and Quintana, 2018). Activation of the AhR affects the expression of immunoregulatory genes and the innate function of macrophages and dendritic cells (DC) (Vogel et al., 2008; Bankoti et al., 2010; Jin et al., 2010; Vogel et al., 2013). The exposure to AhR-activating ligands, including environmental toxicants such as dioxins and polycyclic aromatic hydrocarbons (PAHs) can trigger a dysregulation of cytokines and the development of immune system disorders (Marshall and Kerkvliet, 2010; Rothhammer and Quintana, 2019). Recent reports showed that ambient particulate matter (PM) may contain significant amounts of PAHs, which are a relevant source of environmental AhR ligands (Vogel et al., 2020). Consequently, recent work described a central role of the AhR as a mediator of inflammatory genes induced by air pollution PM and their chemical components generated by traffic and combustion in urban areas or by wildfire smoke (Castañeda et al., 2018; D'Evelyn et al., 2021; Dahlem et al., 2020; Yuan et al., 2020; Young et al., 2021). Further, a recent study revealed that PAHs are a critical component in PM promoting T helper 17 (Th17) cell-polarization and secretion of IL-17A from T cells *in vivo* associated with autoimmune diseases (O'Driscoll et al., 2018). Recently, we reported that TCDD as well as PAH-containing PM directly activate AhR and induce inflammatory markers associated with an enhanced activation of DC responsible for differentiation of naive T cells towards a Th17-like phenotype and an increased production of interleukin 22 (IL-22) (Castañeda et al., 2018). More than a decade ago Francisco Quintana’s and Brigitta Stockinger’s teams showed that the AhR is essential for the production of IL-22 and differentiation of Th17 cells (Quintana et al., 2008; Veldhoen et al., 2009). Further, it became evident that AhR is a key player driving the production of IL-22 in innate lymphoid cells 3 (ILC3) and regulates the development of ILC3 controlling intestinal immunity and inflammation (Lee et al., 2011; Qiu et al., 2012). The expression of IL-22 triggered by IL-21 and IL-23 in Th17 cells was controlled by AhR and RAR-related orphan receptor gamma t (RORγt) (Kiss et al., 2011; Yeste et al., 2014).

More recently the expression of IL-22 was found to be upregulated in DC and macrophages by AhR ligands (Vogel et al., 2013; Ishihara et al., 2019a). Further, we and other research teams revealed that a group of Nuclear Factor-kappa B (NF-κB) target genes encoding cytokines and chemokines may also be regulated by AhR (Kimura et al., 2009; Vogel et al., 2013; Øvrevik et al., 2014; Lahoti et al., 2015; Kado et al., 2017; Domínguez-Acosta et al., 2018; Zhu et al., 2018). NF-κB is a key transcription factor which regulates the transcription of many regulatory cytokines, chemokines, receptors and enzymes in innate immune cells (Hoffmann et al., 2002). Activation of NF-κB is induced through multiple Pattern Recognition Receptor (PRR) pathways such as the Toll-Like receptors (TLRs) (Mogensen, 2009; Kawasaki and Kawai, 2014). Thus, activation of TLR and NF-κB signaling downstream has a fundamental role in regulation of inflammation and disease. Interestingly, the transcriptional regulation of the *Ahr* gene has been found to be controlled by NF-κB subunits RelA and RelB (Vogel et al., 2014; Iu et al., 2017) highlighting the interaction of AhR and NF-κB signaling. Furthermore, AhR can physically interact with RelB as a component of the non-canonical AhR pathway, resulting in cooperative activation of human *Il8* gene transcription (Vogel et al., 2007). In contrast to the canonical AhR pathway, which essentially depends on the heterodimerization of AhR with the Aryl Hydrocarbon Receptor Nuclear Translocator (ARNT) and binding on consensus dioxin responsive elements (DRE), the non-canonical AhR pathway involves the interaction of AhR with other proteins such as the Krüppel-like Factor 6, members of the NF-κB protein family or the cross-talk with estrogen receptor alpha (ERα) (Matthews and Gustafsson, 2006; Vogel et al., 2007; Denison et al., 2011; Vogel et al., 2014; Iu et al., 2017; Wright et al., 2017). The non-canonical AhR pathway also includes ligand-independent activation of AhR signaling as described for cAMP-dependent protein kinase (PKA)-mediated activation of AhR (Oesch-Bartlomowicz et al., 2005; Vogel et al., 2007). The cross-regulation between the NF-κB and AhR signaling axis is of particular importance leading to dysregulation of genes including cytokines and cytochrome P4501A1 (CYP1A1) (Kim et al., 2000; Ke et al., 2001; Sulentic et al., 2004; Vogel et al., 2011; Vogel et al., 2013; Vogel et al., 2014; Salisbury and Sulentic, 2015) which can be an important contributing factor to the pathology of chronic diseases.

IL-22 is primarily produced by lymphoid cells including activated T cells and innate ILC. The production of IL-22 by ILC plays an important role in host defense, mucosal homeostasis, and protection against chronic inflammation (Zenewicz et al., 2008; Wei et al., 2020). On the other hand, models of autoimmunity, psoriasis, and atherosclerosis revealed a pathogenic function of IL-22 (Ma et al., 2008; Geboes et al., 2009; Rattik et al., 2015). Studies with preclinical models also suggest a complex role of IL-22 indicating that the effects of IL-22 are context specific and dependent on the microenvironment, which makes it difficult to predict the impact of IL-22 in disease (Wei et al., 2020). Moreover, IL-22 has been implicated in cancer development and progression (Di Lullo et al., 2015; Hernandez et al., 2018; Keir et al., 2020) including colon cancer (Huber et al., 2012; Kirchberger et al., 2013; Markota et al., 2018; Dmitrieva-Posocco et al., 2019; Arshad et al., 2020) and breast cancer (Kim et al., 2014; Banerjee and Resat, 2016; Irshad et al., 2017; Voigt et al., 2017; Zhang et al., 2020). Especially the dysregulation and prolonged expression of IL-22 seems to be associated with the mechanisms of pathological inflammation and tumorigenesis (Sonnenberg et al., 2011; Lim and Savan, 2014). An increased expression of IL-22 in breast cancer has been found to be associated with elevated infiltration of tumor associated macrophages and poor clinical outcomes (Zhao et al., 2021). This is consistent with the fact that IL-22 induces proliferative and anti-apoptotic signaling pathways and promotes epithelial-to-mesenchymal transition (Perusina Lanfranca et al., 2016). Subsequently, inhibition of IL-22 may be beneficial in cancer, whereas an enhanced IL-22 expression may be favorable in gut infections and inflammatory bowel disease. Based on the pleiotropic role of IL-22 it is important to identify the mechanisms of IL-22 regulation especially in the tumor microenvironment. Besides T cells and ILCs as the main cellular sources of IL-22 production, several myeloid cell subsets including neutrophils, dendritic cells, mast cells and macrophages may secrete IL-22 during inflammation (Ikeuchi et al., 2005; Hansson et al., 2013; Zindl et al., 2013; Chellan et al., 2014; Mashiko et al., 2015; Fumagalli et al., 2016). Because the control of IL-22 expression by AhR signaling, the focus of this study is on the modulatory mechanism induced by AhR ligands in bone marrow-derived macrophages (BMM) in cross-talk with NF-κB in TLR-activated BMM. Here we also investigated the effect of environmental PM derived from traffic related sources and wildfires to activate AhR and IL-22.

## Materials and Methods

### Reagents and Preparation of PM

Dimethyl sulfoxide (DMSO) was purchased from Sigma. 2,3,7,8-tetrachlorodibenzo-p-dioxin (TCDD) (>99% purity) was originally obtained from Dow Chemical Co. (Midland, MI, United States). 6-Formylindolo[3,2-b]carbazole (FICZ), Indole-3-carbinol.

(I3C), 12-O-tetradecanoylphorbol-13-acetate (TPA) and other molecular biological reagents were purchased from Cayman Chemicals (Ann Arbor, MI, United States) and Applied Biosystems (Foster City, CA, United States). TLR ligands were purchased from InvivoGen (San Diego, CA, United States). RelB-specific polyclonal antibody from Active Motif (Carlsbad, CA, United States). AhR-specific polyclonal antibody from Enzo Life Sciences (Farmingdale, NY, United States). The traffic-related air pollution (TRAP)-related PM_2.5_ was collected from an exposure facility immediately adjacent to a major freeway tunnel system in Northern California (Patten et al., 2020) *via* impaction-based filter sampling and extracted according to the protocols of Bein and Wexler (2014) and Bein and Wexler (2015). TRAP samples were collected before and during the Sonoma/Napa wildfire in 2017. The wildfire PM sample was collected during the Carr wildfire in Northern California in 2018. The dry PM extracts were resuspended in DMSO and sonicated immediately prior to treatment of the cells.

### Isolation, and Differentiation of Bone Marrow-Derived Macrophages

Primary bone marrow (BM) progenitor cells were isolated and differentiated from wildtype (WT), AhR null mice (AhR^−/−^), and RelB null mice (RelB^−/−^) as described earlier (Vogel et al., 2013; Castañeda et al., 2018; Ishihara et al., 2019b). C57BL/6 WT mice (age 5–6 weeks) were purchased from the Jackson Laboratory (Sacramento, CA, United States). Initial breeding stocks for B6. AhRtm1Bra (AhR^−/−^) mice were kindly provided by Christopher Bradfield (University of Wisconsin Madison). A breeding of RelB^−/−^ mice was kindly provided by Alexander Hoffmann (University of California Los Angeles). The colonies of AhR^−/−^ and RelB^−^ were backcrossed on a C57BL/6 background and continually maintained at the University of Davis and genotyped using the DNA/RNA Shield reagent (Zymo Research, Irvine, CA, United States). The animals were maintained according to the guidelines set by the University of California Davis. The studies involving animals were reviewed and the protocol for animal care and use was approved and completed by the Institutional Animal Care and Use Committee (IACUC) on January 21, 2021 at the University of California Davis (#21564). This project was conducted in accordance with the ILAR guide for the care and use of laboratory animals, and the UC Davis Animal Welfare Assurance on file with the US Public Health Service.

Briefly, femurs from 8-week old female mice were isolated under sterile conditions, and BM cells were extracted via a Roswell Park Memorial Institute (RPMI) media-loaded syringe. Cells were passed through a 30 μm cell strainer, and the supernatant was centrifuged for 5 min at 1,000 ×*g*. The supernatant was decanted, and the pellet was resuspended and cultured in RPMI medium. Cells from three age-matched female mice were pooled and plated in 12- or 24-well cell culture plates for the various *in vitro* experiments and experimental groups. Differentiation of BM-derived macrophages was performed in the presence of granulocyte-macrophage colony-stimulating factor (GM-CSF; 20 ng/ml; Tonbo Biosciences, San Diego, CA). Differentiation of macrophages occurred over 6 days and adherent macrophages were purified (85–90%) as described previously (Ishihara et al., 2019b). The appropriate media was replenished every 2 days. On day 6, macrophages were treated in 12 well plates at a density of 5 × 10^4^ cells/well and treated in triplicates with TLR and AhR ligands as indicated. At least three independent experiments were performed for the various *in vitro* studies. In order to test environmental pollutants on the expression of IL-22, BMM were treated with PM (10 μg/ml) collected from TRAP and wildfires (WF) in California.

### Isolation and Activation of Naive CD4+ T Cells

CD4^+^CD62L^+^ naive T cells were isolated from C57BL/6 WT mice using Miltenyi Biotec’s mouse CD4^+^naïve T cell isolation kit as described (Castañeda et al., 2018). T cells were activated with MACSiBeads conjugated with CD3ε and CD28T antibodies and cultured at a 1:1 ratio with T cells. CD4^+^CD62L^+^ naive T cells were isolated and treated with TCDD (1 nM) and IL-21 (30 ng/ml). RNA was isolated to analyze IL-22 gene expression.

### RNA Isolation and Quantitative Real-Time PCR

The preparation of RNA and qPCR was performed according to the manufacturer’s protocol (Zymo Research and LightCycler 480, Roche) and as described earlier (Vogel et al., 2013). Total RNA was isolated from cells using a Quick-RNA Mini prep isolation kit (Zymo Research), and cDNA synthesis was performed using a cDNA synthesis kit Applied Biosystems (Foster City, CA, United States). Detection of β-actin and differentially expressed target genes was performed with a LightCycler LC480 Instrument (Roche Diagnostics, Indianapolis, IN, United States) using the Fast SYBR Green Master Mix (Applied Biosystems) according to the manufacturer’s instructions. The primers for each gene were designed on the basis of the respective cDNA or mRNA sequences using OLIGO primer analysis software provided by Steve Rozen and the Whitehead Institute/Massachusetts Institute of Technology Center for Genome Research so that the targets were 100–200 bp in length. To confirm the amplification specificity, the PCR products were subjected to melting curve analysis.

### Measurement of IL-22 mRNA Half-Life

To determine if TCDD and LPS affects the IL-22 mRNA half-life, actinomycin D (5 μg/ml) was added to cells after 24 h of stimulation with TCDD and LPS. BMM were then rinsed with PBS and culture media containing actinomycin D was added. Cells were incubated for an additional 0.5–4 h in standard culture conditions. RNA was isolated from cells at the indicated time points after the addition of actinomycin D, and qPCR was performed as described above. Results were depicted as the percent of RNA remaining *versus* the time after the addition of actinomycin D (hour 0).

### Measurement of IL-22 Concentrations in BMM

Cell culture supernatants from BMM cultures were used to quantify IL-22 protein levels using BioLegend’s (San Diego, CA, United States) mouse ELISA kit according to the manufacturer’s protocol and as described previously (Castañeda et al., 2018). Cell-free conditioned medium was obtained by centrifugation (300 g, 10 min).

### Cloning of the Mouse IL-22 Promoter, Site-Directed Mutagenesis, and Transfection Experiments

Transient transfection and luciferase reporter studies using IL-22 promoter constructs were performed as described (Vogel et al., 2007; Vogel et al., 2014). The luciferase reporter construct containing the IL-22 promoter sequence was provided by SwitchGear Genomics (Menlo Park, CA, United States) corresponding to a −3,455 bp construct of the mouse promoter sequence. DNA promoter analysis and identification of putative transcription factor binding sites of the mouse IL-22 gene was performed using the TFSEARCH program (Heinemeyer et al., 1998). The mutations of the NF-κB, the DRE, and RelBAhRE sequences in the mouse IL-22 gene promoter were carried out by site-directed mutagenesis (Stratagene, La Jolla, CA) as described previously (Vogel et al., 2007; Vogel et al., 2014). The NF-κB luciferase reporter was a kind gift of Courtney Sulentic (Wright State University, OH). The plasmid DRE and NF-κB reporter constructs were amplified and purified with Zymo PURE-Endo Zero plasmid isolation kit (Zymo Research). Transfection of plasmid DNA and short interfering RNA (siRNA) into BMM was performed via Nucleofector technology as described (Vogel et al., 2014). Briefly, 10^6^ BMM were resuspended in 100 μl of Nucleofector Solution V (Amaxa GmbH, Köln, Germany) and nucleofected with 1.0 μg of plasmid DNA or siRNA using program V-001, which is preprogrammed into the Nucleofector device (Amaxa GmbH).

### Chromatin Immunoprecipitation Assays

ChIP assays with AhR- and RelB-specific antibodies were analyzed by PCR using primer pairs covering the specified DRE and RelBAhRE regions of mouse *IL-22* as depicted in Figure 6A. Genomic DNA and the input DNA were separated by agarose gel electrophoresis. ChIP assay samples from BMM were analyzed as described (Vogel et al., 2014; Vogel et al., 2019a). In brief, BMM cells were treated with TCDD and LPS for the indicated times and protein-DNA complexes were cross-linked with 1% formaldehyde for 10 min and prepared for ChIP assay. DNA was purified using a DNA purification kit (Zymo Research) and eluted in 50 μl. ChIP DNA was amplified by real-time PCR with primers covering the specified region DRE and RelBAhRE of the IL-22 promoter.

### Statistical Methods

Data are expressed as mean ± standard error of the mean (SEM). Inter-group (wildtype vs. RelB^−/−^ or AhR^−/−^) comparisons were performed using two-way ANOVA followed by Bonferroni’s post-test. Intra-group comparisons (within the wildtype treatment groups) were assessed by one-way ANOVA followed by *post hoc* Tukey’s multiple comparison test using GraphPad PRISM 9 software. A value of *p <* 0.05 was considered statistically significant.

## Results

### The Time-dependent Effects of AhR and TLR4 Activation on IL-22

**FIGURE 1:**
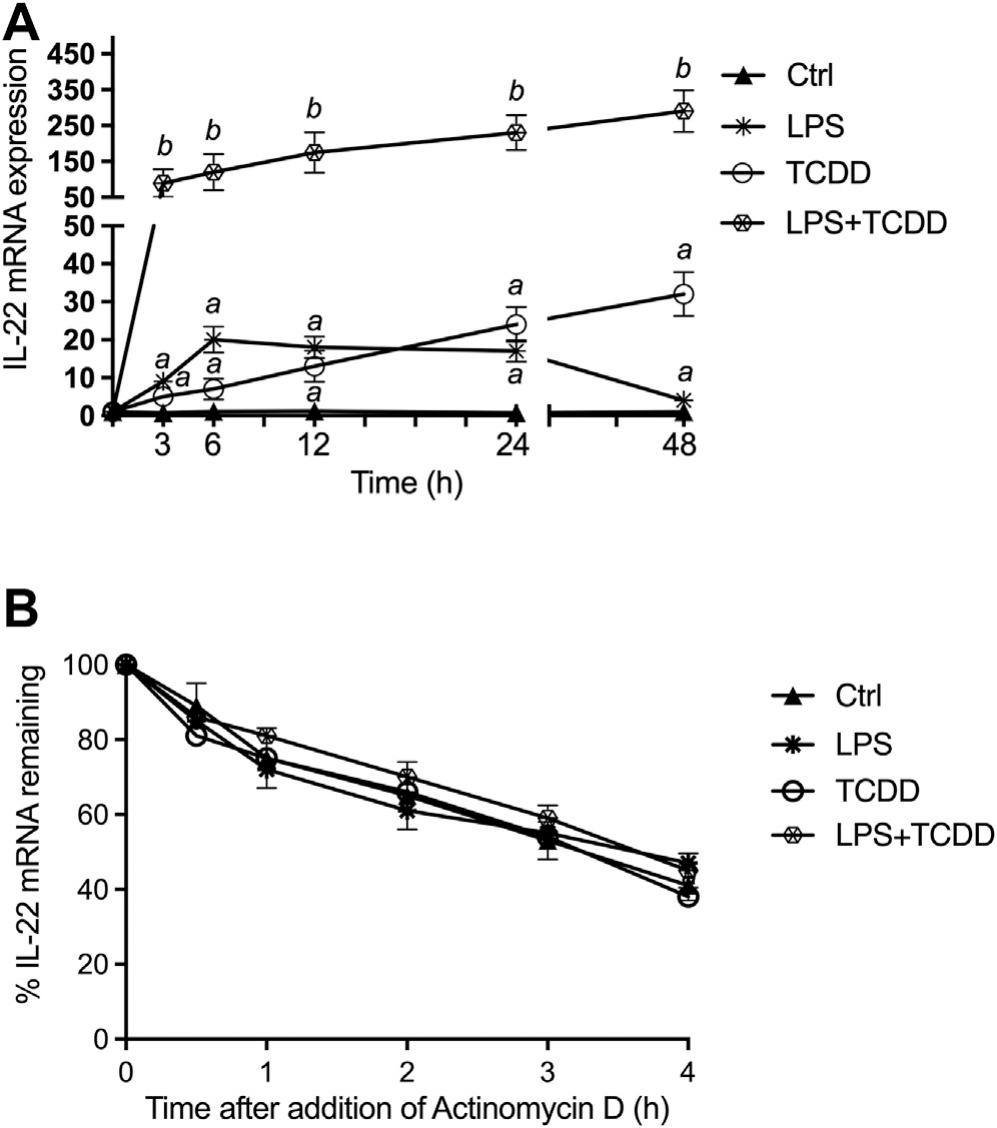
Synergistic effect of AhR and TLR4 activation on IL-22 mRNA expression. **(A)** Synergistic effect of TCDD and LPS on time-dependent expression of IL-22 in BMM. BMM derived from 8-week old female B6 wt mice were treated with LPS (1.0 μg/ml), TCDD (1 nM), or co-treated with TCDD plus LPS for 3–48 h. The expression of IL-22 was analyzed using qPCR. The expression was corrected against the housekeeping gene ß-actin. Results are presented as mean ± SEM of triplicates from three independent experiments and the *y*-*axis* represents mRNA expression level as fold increase above control. Lowercase letters indicate significant differences between control and treatment groups. ^*a*^significantly higher than control, *p* < 0.05; ^*b*^ significantly higher than cells treated with TCDD or LPS alone, *p* < 0.05 **(B)** Effect of actinomycin D on IL-22 mRNA levels in BMM. BMM from WT mice were cultured with TCDD plus LPS for 24 h before the addition of actinomycin D (5 μg/ml). Cells were harvested for RNA extraction and IL-22 expression analysis via qPCR at the times indicated. Expression of IL-22 mRNA is normalized to the relative expression ratio at the time of addition of actinomycin D (hour 0). Results are presented as mean ± SEM of triplicates from three independent experiments.

The time-dependent effects of AhR and TLR4 activation by TCDD and LPS, respectively, on IL-22 expression in BMM derived from C57BL/6 wildtype (WT) mice are shown in Figure 1A. TCDD induced IL-22 mRNA expression by 5-fold above control after 3 h treatment and continued to increase over time to a maximum level of 32-fold at 48 h after treatment with TCDD (Figure 1A). LPS rapidly increased IL-22 mRNA 9-fold after 3 h treatment with a maximum level of 20-fold above control at 6 h. In contrast to LPS alone, the addition of the AhR ligand TCDD significantly increased the expression of IL-22 in LPS-activated BMM and led to a sustained elevated level of 290-fold at 48 h.

### Effect of TCDD and LPS on IL-22 mRNA Stability

The modification of IL-22 mRNA stability could be one possible mechanism for the increase of IL-22 mRNA expression induced by LPS and TCDD. We then evaluated IL-22 mRNA half-life in TCDD and LPS treated WT BMM, using actinomycin D, an inhibitor of transcription (Figure 1B). Results showed that neither the single treatment with TCDD or LPS nor the combinatorial treatment modifies the half-life of IL-22 mRNA, compared to the half-life measured in control cells, which is about 3.5 h. Results of these analyses indicated that the induction of IL-22 mRNA by TCDD and LPS is not due to a stabilization of its mRNA.

### Activation of IL-22 by Various AhR Ligands

**FIGURE 2:**
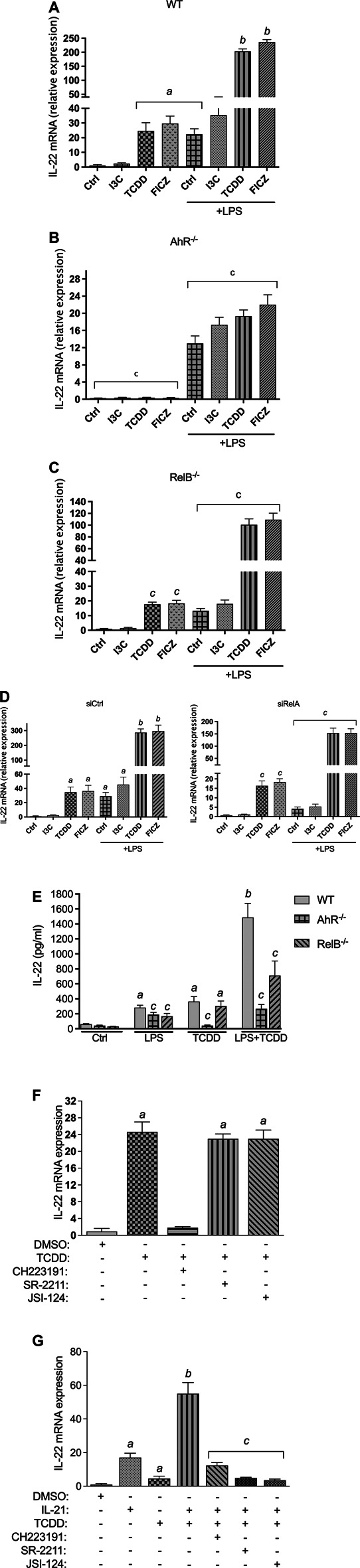
Effect of AhR ligands on IL-22 mRNA expression. BMM derived from **(A)** WT, **(B)** AhR^−/−^, and **(C)** RelB^−/−^ mice were treated with I3C (50 μM), TCDD (1 nM), and FICZ (100 nM), in non-activated and LPS- (1.0 μg/ml) activated BMM for 24 h. The expression was corrected against the housekeeping gene ß-actin. Results are presented as mean ± SEM and the *y*-*axis* represents mRNA expression level as fold increase above control. **(D)** Effect of gene silencing of RelA on the expression of IL-22. BMM derived from WT mice were transfected with scrambled siRNA as control or siRNA specific for RelA for 24 h and then treated with I3C (50 μM), TCDD (1 nM), and FICZ (100 nM), in non-activated and LPS- (1.0 μg/ml) activated BMM for 24 h. **(E)** BMM derived from WT, AhR^−/−^, and RelB^−/−^ mice were stimulated with TCDD in presence or absence of LPS. After 24 h, IL-22 production was determined in cell supernatant by ELISA. **(F)** Effect of TCDD on IL-22 expression in BMM is independent of RORγt and JAK/STAT. BMM were pre-treated with antagonists of AhR (CH223191, 10 μM), antagonists of RORγt (SR-2211, 5 μM) and the JAK/STAT inhibitor JSI-124 (5 μM) for 10 min and then treated with 1 nM TCDD for 24 h. **(G)** Effect of TCDD on IL-22 mRNA expression in CD4^+^ T cells depends on RORγt and JAK/STAT. CD4^+^ T cells were stimulated with IL-21 (30 ng/ml) for 24 h in presence or absence of TCDD (1 nM). To inhibit AhR, RORγt and JAK/STAT, CD4^+^ T cells were pre-treated with antagonists of AhR (CH223191, 10 μM), antagonists of RORγt (SR-2211, 5 μM) and the JAK/STAT inhibitor JSI-124 (5 μM) for 10 min and then treated with 1 nM TCDD for 24 h. Cells were harvested for RNA extraction and IL-22 expression analysis via qPCR. Results are presented as mean ± SEM of triplicates from three independent experiments. Lowercase letters indicate significant differences between control and treatment groups. ^*a*^significantly higher than control, *p* < 0.05; ^*b*^significantly higher than AhR ligands or LPS alone, *p* < 0.05, ^*c*^significantly lower than BMM derived from wt mice, *p* < 0.05.

Expression analysis using BMM derived from WT showed that I3C, TCDD, and FICZ induced the expression of IL-22 mRNA in steady state BMM (Figure 2A). Treatment with I3C led to a small, but statistically not significant increase of IL-22 after 24 h, followed by a 24- and 29-fold increase by TCDD and FICZ, respectively. TCDD and FICZ significantly increased the expression of IL-22 in LPS-activated BMM compared to BMM treated with LPS alone (Figure 2A). The induction of IL-22 by AhR ligands in steady-state as well as LPS-activated BMM was AhR-dependent as shown in BMM derived from AhR^−/−^ mice (Figure 2B). The LPS-mediated induction of IL-22 was in part dependent on the presence of RelB as shown in BMM derived from RelB^−/−^ mice (Figure 2C). The synergistic effects of TCDD and FICZ on LPS-induced expression was also significantly lower in RelB^−/-^ BMM compared to BMM derived from WT mice. Further, we considered the role of RelA in AhR- and NF-κB-induced expression of IL-22. Results from transfection experiments for gene silencing of RelA via siRNA showed a reduced induction of IL-22 by TCDD and FICZ and a significant repression of the LPS-mediated induction of IL-22 compared to BMM transfected with a scrambled siRNA (Figure 2D).

Next, we measured the level of IL-22 protein in the supernatant of LPS- and TCDD-treated BMM. Analysis of IL-22 secretion stimulated by AhR ligand TCDD and the TLR4 ligand LPS for 48 h indicate that the induced expression of IL-22 mRNA resulted in an elevated level of the IL-22 protein in supernatant of BMM from WT mice (Figure 2E). The co-treatment of TCDD plus LPS led to a significant increase of IL-22 in the supernatant of BMM derived from WT mice. The analysis of IL-22 production in BMM derived from AhR^−/−^ and Relb^−/−^ mice confirm the important role of AhR and RelB in IL-22 production stimulated by TCDD and LPS treatment (Figure 2E).

### TCDD-Induced Expression of IL-22 in BMM is Independent of RORγt and STAT3

Previous studies have shown that IL-21 stimulates the production of IL-22 in CD4^+^ T cells which involved activation of STAT3, RORγt and the interaction with AhR (Yeste et al., 2014). Here we tested the role of RORγt and STAT3 in the AhR-mediated induction of IL-22 in BMM. The results showed that pre-treatment with an inhibitor of RORγt (SR-2211) had no effect on the expression of IL-22 induced by TCDD (Figure 2F). Further, the pretreatment of BMM with JSI-124, a selective inhibitor of the JAK2/STAT3 signaling pathway, did not affect the expression of IL-22 induced by TCDD. Confirming the data with BMM from AhR^−/−^ mice, pre-treatment with the AhR antagonist CH223191 significantly suppressed the TCDD-triggered expression of IL-22. In addition to BMM, we tested the effect of TCDD on the expression of IL-22 in CD4^+^ T cells. The results showed that inhibition of RORγt and STAT3 repressed the TCDD-stimulated expression of IL-22 in CD4^+^ T cells stimulated with IL-21 (Figure 2G) confirming previous studies (Kiss et al., 2011; Yeste et al., 2014).

### Effect of TCDD on IL-22 Expression in TLR-Activated BMM

**FIGURE 3:**
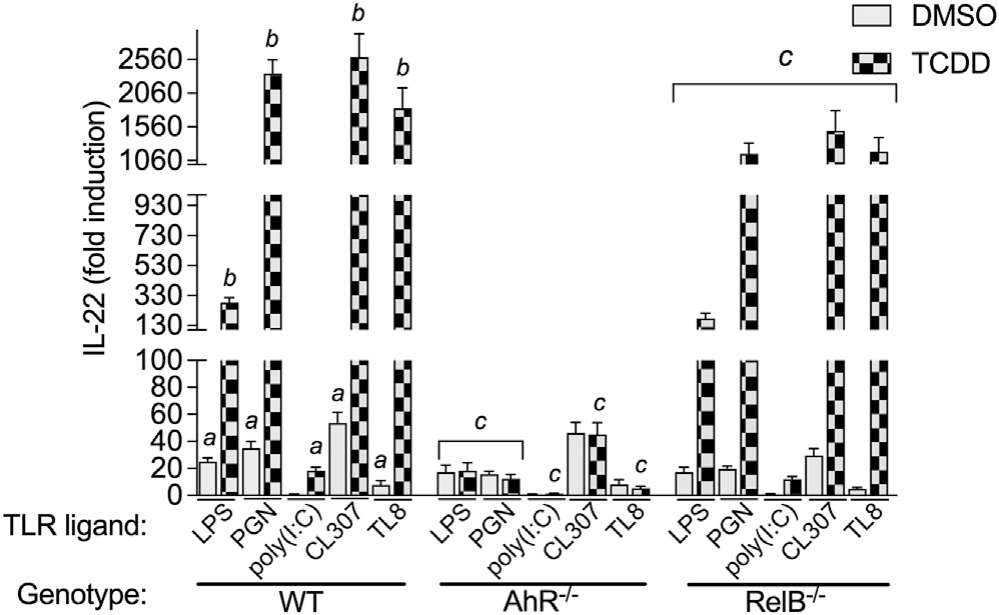
Effect of TLR ligands on IL-22 mRNA expression in macrophages. BMM derived from WT, AhR^−/−^, and RelB^−/−^ mice were treated with LPS (1.0 μg/ml), PGN (1.0 μg/ml), poly(I:C) (1.0 μg/ml), and CL307 (1.0 μg/ml), in non-treated and TCDD- (1 nM) treated BMM. Non-treated cells received 0.1% DMSO as vehicle control. The expression of IL-22 was analyzed using qPCR. The expression was corrected against the housekeeping gene ß-actin. Results of triplicates from three independent experiments are presented as mean ± SEM and the *y*-*axis* represents mRNA expression level as fold increase above control. Lowercase letters indicate significant differences between control and treatment groups. ^*a*^significantly higher than control, *p* < 0.05; ^*b*^significantly higher than TLR ligands alone, *p* < 0.05, ^*c*^significantly lower than BMM derived from WT mice, *p* < 0.05.

Here we tested the effect of TCDD in BMM activated by different TLR ligands. We treated BMM with specific TLR ligands including LPS (TLR4), PGN (TLR2), polyinosinic-polycytidylic acid (poly(I:C)) (TLR3), CL307 (TLR7), and TL8-506 (TLR8) for 24 h. BMM were treated with TLR ligands in presence or absence of TCDD. As for LPS, ligands of TLR2, TLR7 and TLR8 induced the expression of IL-22 in WT BMM after stimulation for 24 h (Figure 3). Activation of TLR7 by CL307 led to the strongest induction (53-fold) of IL-22 followed by PGN (35-fold), LPS (25-fold), and TL8-506 (8-fold). The TLR3 ligand poly(I:C) did not change the expression of IL-22 and had no synergistic effect in combination with TCDD. TCDD in cotreatment with poly(I:C) induced IL-22 expression (18-fold) in WT BMM which was lower than the effect of TCDD alone as shown in Figure 2A. The highest synergistic induction of IL-22 in presence of TCDD was found with CL307 (2593-fold) followed by PGN (2353-fold), TL8-506 (1833-fold), and LPS (283-fold).

### Effect of PM Samples on IL-22 Expression and the Activity of AhR and NF-κB

**FIGURE 4:**
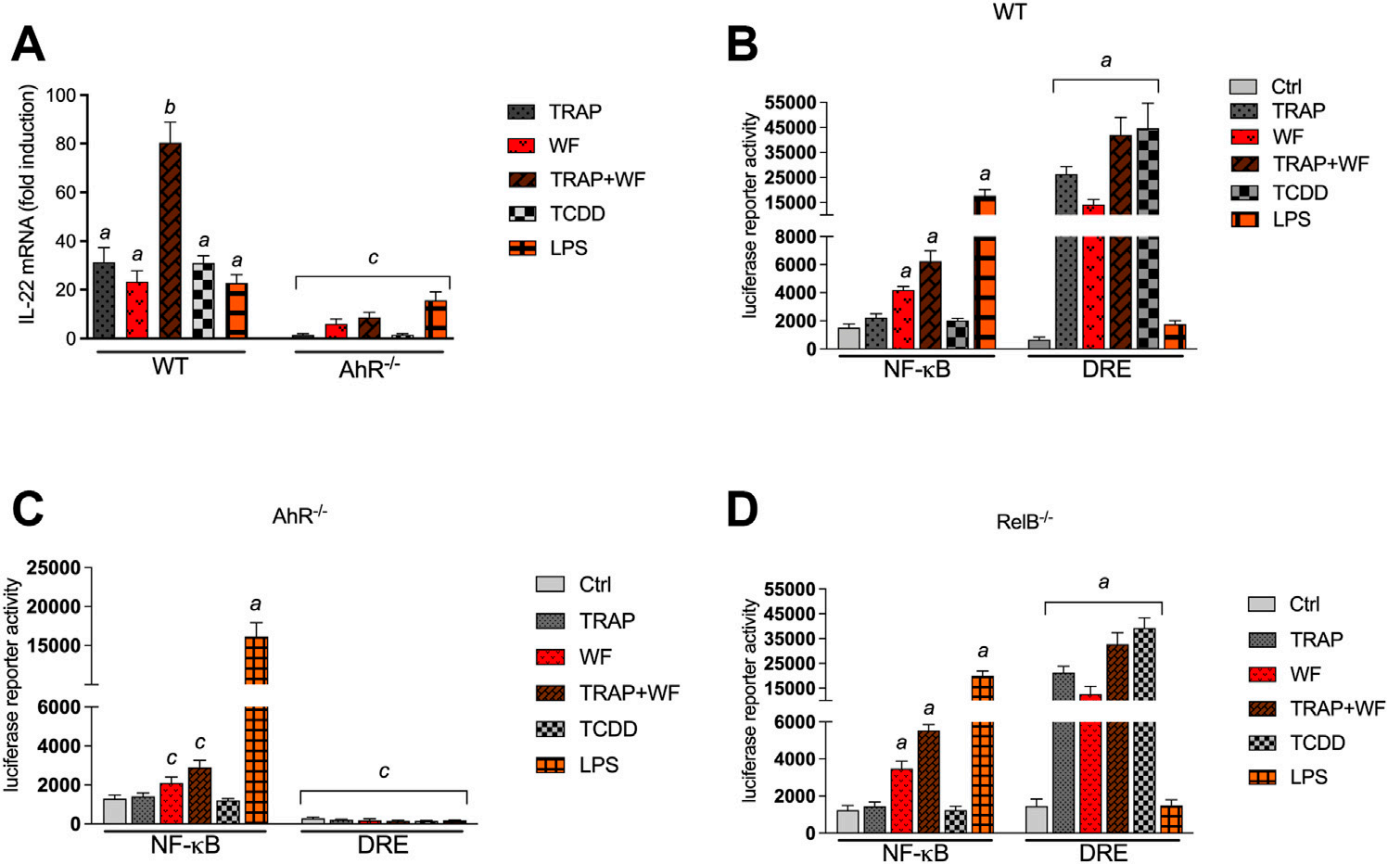
Effect of environmental PM samples on IL-22 mRNA expression and the activity of AhR and NF-κB. **(A)** Effect of wildfire and TRAP PM samples on IL-22 mRNA expression in BMM derived from WT and AhR^−/−^ mice. BMM were treated with 10 μg/ml of particulate matter (PM) samples collected from TRAP and the 2018 Carr wildfire (WF) in California. TRAP samples were collected at two different timepoints in March 2017 before (TRAP) and in September 2017 during the Napa/Sonoma wildfire (TRAP/WF). The expression of IL-22 was analyzed after 24 h using qPCR. The expression was corrected against the housekeeping gene ß-actin. Results of triplicates from three independent experiments are presented as mean ± SEM and the *y*-*axis* represents mRNA expression level as fold increase above control. **(B)** Activation of a NF-κB- and DRE-luciferase reporter activity after exposure to 10 μg/ml PM fractions from TRAP. **(B)** WT BMM, **(C)** AhR^−/−^ BMM and **(D)** RelB^−/−^ BMM were transiently transfected with NF-κB and DRE reporter constructs for 24 h and treated with PM for 4 h. Relative luciferase activity units are given as mean values of triplicates as a result of three independent experiments. Cells were treated with LPS (1.0 μg/ml) and TCDD (1 nM) as positive control. Non-treated cells received 0.1% DMSO as vehicle control. Lowercase letters indicate significant differences between control and treatment groups. ^*a*^significantly higher than control, *p* < 0.05; ^*b*^significantly higher than LPS or TCDD treated cells, *p* < 0.05, ^*c*^significantly lower than BMM derived from WT mice, *p* < 0.05.

In recent years studies have shown that the AhR acts as an environmental sensor of environmental pollutants such as ambient air particulate matter (PM) modifying the immune response (Vogel et al., 2020). Especially, PM collected from TRAP and wildfires have been found to activate the AhR pathway and induce CYP1A1 as well as inflammatory cytokines (Castañeda et al., 2018; Young et al., 2021; O'Driscoll et al., 2018). Here we tested the effects of TRAP-derived PM collected in a tunnel system near San Francisco (CA) before and during a firestorm in 2017 and a PM sample collected from the Carr wildfire in Redding (CA) on the expression of IL-22 in BMM (Figure 4A). All PM samples tested increased the mRNA level of IL-22. Interestingly, a strong induction of 80-fold of IL-22 was found in BMM treated with a PM sample which was collected in the tunnel system during a wildfire (TRAP + WF) in 2017 in Napa/Sonoma (CA). The induction of the TRAP + WF PM was significantly higher compared to the TRAP PM sample collected before the wildfire or the PM sample collected from a wildfire. The TRAP and wildfire PM samples activated the AhR activity as determined in a DRE luciferase assay (Figure 4B). Furthermore, the wildfire PM sample and the TRAP + WF PM sample collected during the wildfire stimulated the NF-κB activity which was less induced by TRAP-derived PM. Further, we tested the contribution of AhR and RelB to PM-induced effects and performed DRE and NF-κB luciferase assays using BMM from AhR^−/−^ and RelB^−/−^ mice. The luciferase activity of NF-κB induced by the wildfire PM sample and the TRAP + WF PM sample collected during a wildfire was significantly reduced in AhR deficient BMM compared to WT BMM (Figure 4C). This indicates that activation of NF-κB by the PM samples is mediated at least in part through AhR signaling supporting the regulation of NF-κB by AhR and the interaction of both signaling pathways as shown in previous studies (Puga et al., 2000; Tian et al., 2002; Øvrevik et al., 2014). The PM samples derived from TRAP and wildfire or the positive control TCDD did not increase DRE luciferase activity in AhR^−/−^ BMM. The results with RelB deficient BMM showed no significant change of PM- or LPS-induced NF-κB activity compared to WT BMM (Figure 4D). A small but statistically not significant decrease in PM- and TCDD-induced DRE activity was observed in RelB deficient BMM compared to WT BMM.

### Compositional Intercomparison of the NorCal Wildfire and TRAP PM Samples

**FIGURE 5:**
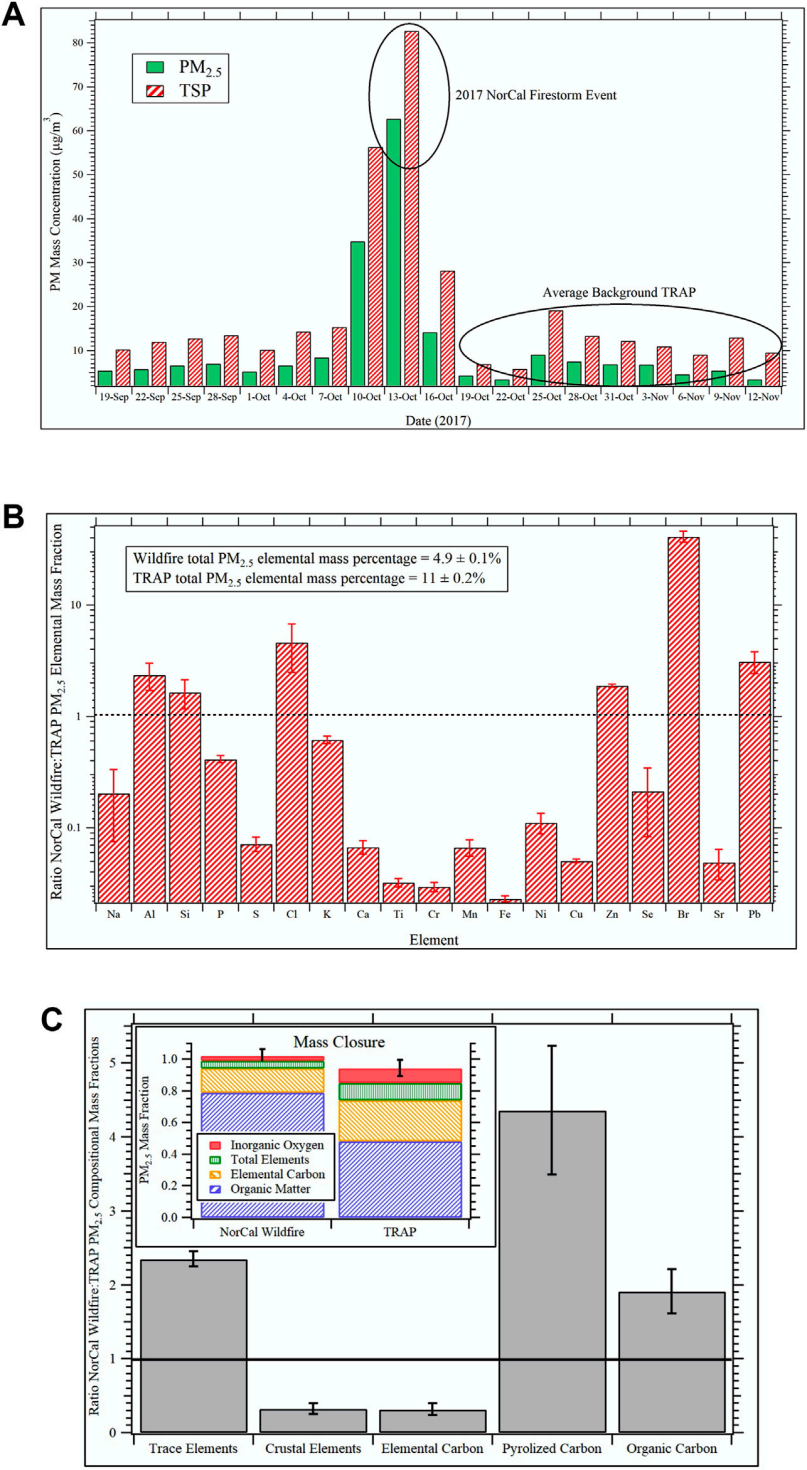
Compositional intercomparison of the NorCal wildfire and TRAP PM samples **(A)** Daily average PM_2.5_ and TSP concentrations determined via gravimetric analyses of filter samples collected at the Caldecott Tunnel Exposure Facility before, during, and after the impact of the 2017 Northern California (NorCal) Firestorm from October 10th through the 16th. For the *in vitro* studies, the TSP filter sample from October 13th was used for the wildfire exposure group while TSP collected continuously from October 19th through November 12th was used for the TRAP exposure group. **(B)** Fractional shift in dose normalized PM_2.5_ elemental composition during the 2017 NorCal Firestorm event relative to the average background TRAP at the Caldecott Tunnel Exposure Facility. Values greater than unity—depicted by black dotted line—indicate the element is enhanced in the wildfire sample relative to TRAP and vice versa for values less than one. Error bars are 99% confidence intervals. **(C)** Fractional shift in dose normalized PM_2.5_ composition distribution as a function of chemical component during the 2017 NorCal Firestorm event relative to the average background TRAP at the Caldecott Tunnel Exposure Facility. Values greater than unity—depicted by the horizontal black line—indicate the chemical component is enhanced in the wildfire sample relative to TRAP and vice versa for values less than one. The inset shows the relative composition distribution for the wildfire sample versus the TRAP background from mass closure analysis. All error bars represent 99% confidence intervals.

The Caldecott Tunnel Exposure Facility (CTEF) is located immediately adjacent to a major freeway tunnel system in the San Francisco Bay Area and facilitates chronic exposure studies on TRAP (Patten et al., 2020; Patten et al., 2021; Edwards et al., 2020). Emissions are drawn directly from the tunnel and delivered unaltered in real-time to onsite exposure chambers. The CTEF is also equipped with an air quality measurement laboratory for continuous emissions monitoring and PM and gas sampling for subsequent offline chemical and toxicological analysis. During the 2017 Northern California (NorCal) Firestorm, the CTEF was heavily impacted by wildfire emissions for a period of approximately 6 days. Figure 5A shows the daily average PM_2.5_ and total suspended particulate (TSP) concentrations determined from gravimetric analyses of 24-hr filter samples collected at the CTEF before, during, and after the wildfire event. The peak impact of wildfire emissions was observed on October 13th when PM_2.5_ concentrations were an order of magnitude larger than the average background TRAP concentrations before and after the event. TSP filter samples from this day were extracted according to protocols described elsewhere (Bein and Wexler, 2014; Bein and Wexler, 2015) and the PM extracts used for the exposure in the *in vitro* studies. Similarly, TSP sampled continuously for the period after the wildfire event from October 19th through November 12th was extracted identically and used for the TRAP exposure.

For compositional intercomparison of the NorCal wildfire and TRAP TSP samples used in the *in vitro* studies, 24-hr PM_2.5_ filter samples collected at the CTEF during the same time periods—one 24-hr sample from October 13th for the wildfire event and four separate 24-hr samples spanning the period October 19th through November 12th for TRAP—were analyzed for elemental composition via X-ray fluorescence (XRF) and elemental carbon and organic carbon (EC/OC) *via* thermal optical reflectance (TOR) according to protocols detailed elsewhere (Berg et al., 2020).

Figure 5B shows the fractional shift in dose normalized PM_2.5_ elemental composition during the 2017 NorCal Firestorm event relative to the TRAP average at the CTEF. Values greater than unity—depicted by the black dotted line—indicate elements that are enhanced in the wildfire sample relative to TRAP and vice versa for values less than one. The error bars represent 99% confidence intervals. Analyzed elements not included in the figure are magnesium, vanadium, rubidium, and zirconium (detected in TRAP but not the wildfire sample) and arsenic (not detected in TRAP or wildfire samples). Significant enhancements in aluminum, silicon, chlorine, zinc, bromine, and lead were observed in the wildfire sample while TRAP was enhanced in crustal elements associated with road dust. The most striking difference is the 40-fold increase in bromine, followed by a 5-fold increase in chlorine and 3-fold increase in lead (Figure 5B). Given the NorCal firestorm decimated entire neighborhoods throughout Napa and Sonoma Counties, it is posited that the bromine and chlorine enhancements are associated with polyhalogenated organics like biphenyls, diphenyl ethers, and dibenzo dioxins and furans from combustion of synthetic materials in the built environment (Lemieux et al., 2004). Lead, on the other hand, is ubiquitous throughout the environment due to years of leaded gasoline and lead pipes and was likely vaporized during the fire and then nucleated or condensed on preexisting PM upon dilution.

The fractional shift in dose normalized PM_2.5_ composition distribution as a function of chemical category during the NorCal Firestorm event relative to the TRAP average at the CTEF is shown in Figure 5C. Again, values greater than unity (black horizontal line) indicate an enhancement of the component in the wildfire sample relative to TRAP and vice versa for values less than one. The inset of Figure 5C shows results from mass closure analysis of the wildfire and TRAP samples on a mass fraction of total PM_2.5_ basis. A value of unity indicates that the sum of components analyzed fully account for total PM_2.5_ mass, while lower and higher values indicate un-apportioned and over-apportioned mass, respectively. All error bars represent 99% confidence intervals. Composite sums for the wildfire and average TRAP samples are 1.02 ± 0.04 and 0.94 ± 0.05, respectively.

In addition to the trends in trace versus crustal elements noted previously, the wildfire sample is enhanced in organic carbon relative to TRAP by a factor of 2 (Figure 5C). Pyrolyzed carbon is an artifact of the TOR measurement where certain organic molecules undergo pyrolysis during the temperature ramp and thus get measured as elemental carbon. Data are then corrected by subtracting this from the EC value and adding it to the OC value. The relevance here is that it indicates significant differences in the nature of the organic carbon between the wildfire and TRAP samples, which likely accounts for differences in toxicity, especially given the bromine and chlorine enhancements discussed previously. Wildfires are known to produce large amounts of brown carbon (Palm et al., 2020), and this may have an impact on the pyrolyzed carbon artifact as well. Vehicular emissions and wildfires are both well-known sources of EC but for wildfires, this depends heavily on combustion phase with flaming tending to produce more EC than smoldering (Andreae and Merlet, 2001). The significant depletion in wildfire EC relative to TRAP suggests the sampled plume was dominated by smoldering combustion, which also tends to produce significantly larger amounts of organic carbon.

### Regulation of IL-22 Promoter Activity by AhR

**FIGURE 6:**
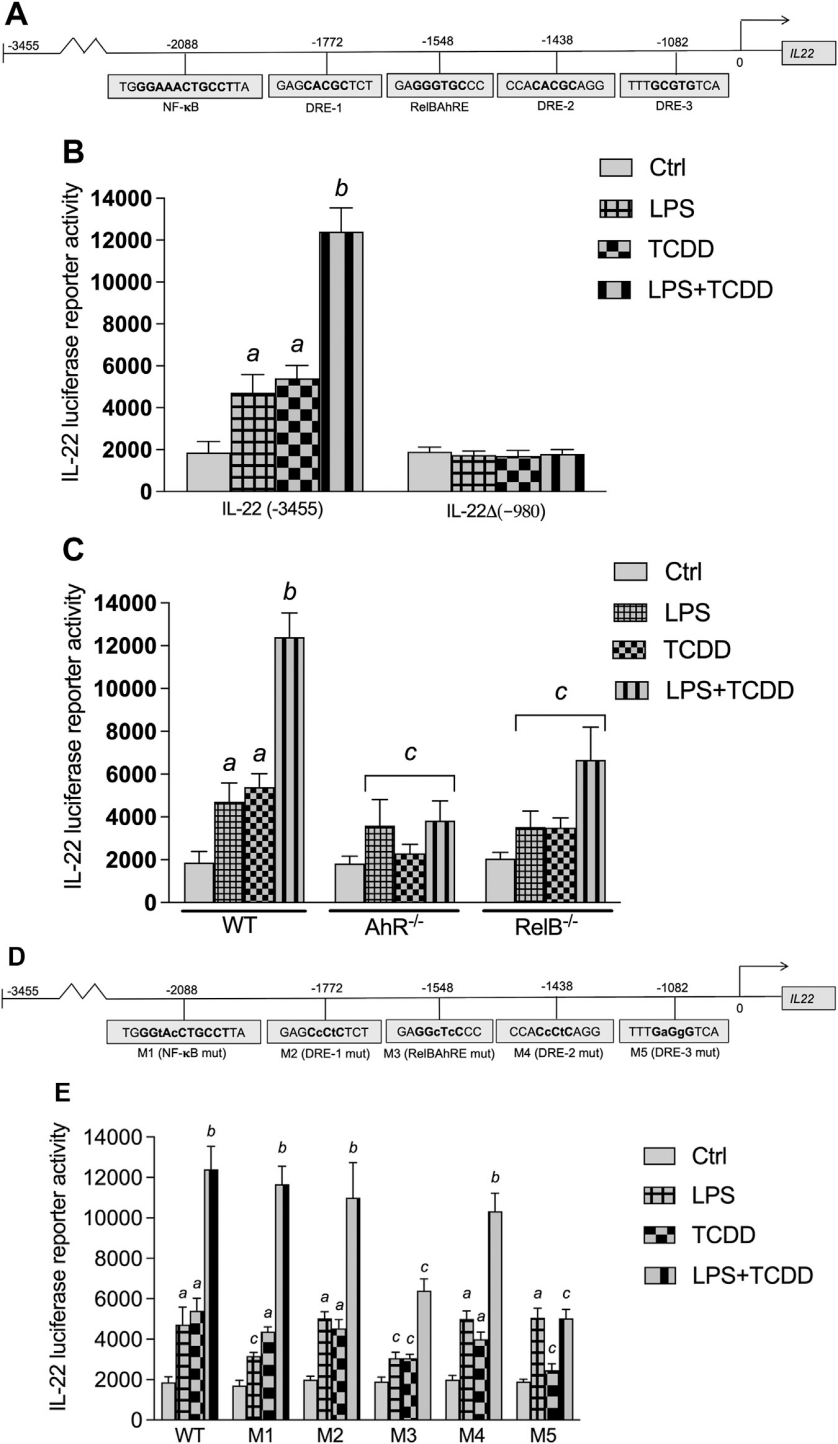
Effect of AhR and TLR4 activation on IL-22 promoter activity. **(A)** Schematic illustration of the mouse IL-22 promoter containing 3,455 bp upstream of the transcriptional start site. Positions of putative DRE, RelBAhRE, and NF-κB DNA binding sites are presented. **(B)** Effect of TCDD and LPS on IL-22 promoter activity. BMM derived from WT mice were transiently transfected with the full-length IL-22 or a IL-22 deletion construct containing 980 bp upstream of the start site. **(C)** BMM derived from WT, AhR^−/−^ and RelB^−/−^ mice were transiently transfected with the full-length IL-22 construct containing 3,455 bp upstream of the start site. Cells were treated with 1 nM TCDD and 1.0 μg/ml LPS or co-treated with LPS plus TCDD for 24 h. **(D)** Schematic illustration of the mutated (indicated by *small letters*) NF-κB (M1), DRE-1 (M2), RelBAhRE (M3), DRE-2 (M4), and DRE-3 (M5) sites of the mouse *Il22* gene. **(E)** Effect of LPS and TCDD on mutation constructs of the mouse IL-22 promoter. BMM were transiently transfected with the full-length (wt) and the mutation constructs M1 to M5 and treated with 1 nM TCDD and 1.0 μg/ml LPS or co-treated with LPS plus TCDD for 24 h. Mean +S.D. of triplicates from three independent experiments are given. Lowercase letters indicate significant differences between control and treatment groups. ^*a*^significantly higher than control, *p* < 0.05; ^*b*^significantly higher than TCDD or LPS alone, *p* < 0.05; ^*c*^significantly lower than BMM derived from WT mice, *p* < 0.05.

Three DREs, one RelB/AhR responsive element (RelBAhRE), and one consensus NF-κB site within 3,455 bp upstream of the transcriptional start site of the mouse *Il22* gene were identified (Figure 6A). A promoter construct containing 3,455 bp of the regulatory region of the mouse *Il22* gene was used for transient transfection in BMM. LPS and TCDD increased the IL-22 promoter activity, which was further increased by co-treatment with LPS plus TCDD (Figure 6B). Next, we created a 980 bp deletion construct which did not contain the DRE, NF-κB or RelBAhRE binding elements as identified above. Treatment with TCDD or LPS had no effect on IL-22 promoter activity of the 980 bp deletion construct suggesting the requirement of the NF-κB, DRE and RelBAhRE binding sites to mediate the induction by TCDD and LPS. Transfection of the full-length IL-22 construct using BMM derived from AhR^−/−^ mice indicated that AhR is required to mediate the induction of IL-22 by TCDD and the synergistic effect stimulated by the co-treatment with TCDD plus LPS (Figure 6C). The results from transfection experiments with BMM derived from RelB^−/−^ mice suggest that the effects of TCDD and LPS at least in part depend on the function of RelB. In order to test the relevance of the NF-κB, DRE and RelBAhRE binding sites, mutation constructs of the IL-22 promoter were generated as schematically shown (Figure 6D). Mutation of the NF-κB site (M1) lowered the stimulation of the promoter activity induced by LPS, but not TCDD (Figure 6E). The synergistic effect induced by the co-treatment with LPS and TCDD on IL-22 promoter activity was not significantly affected by mutation of the NF-κB site. The TCDD and LPS induced IL-22 activity was not affected by mutations of the DRE-1 (M2) or DRE-2 (M4) binding sites. However, the mutation of the RelBAhRE site (M3) caused a lower induction of IL-22 promoter activity stimulated by LPS and TCDD. The TCDD-induced promoter activity of IL-22 was also repressed with the promoter construct containing a mutation of the DRE-3 (M5) consensus element at −1,082 bp closer to the start site of the *Il22* gene. The results suggest that the NF-κB site is involved in the LPS stimulation of IL-22, however, the DRE-3 and RelBAhRE binding sites are critical to mediate the synergistic effects of LPS and TCDD co-treatment.

### Enhanced Recruitment of AhR and RelB to the DRE and RelBAhRE Binding Sites of the IL-22 Promoter

**FIGURE 7:**
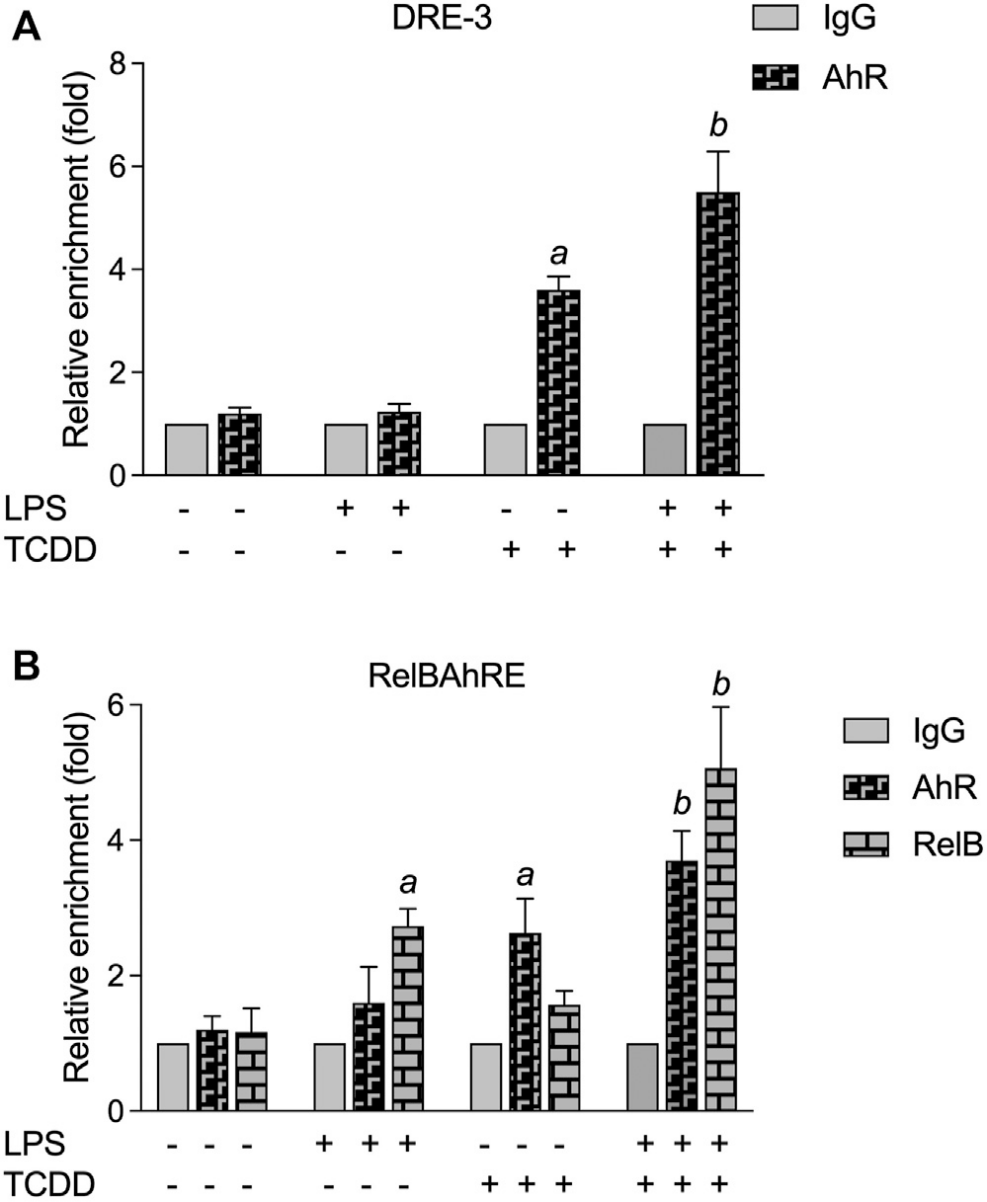
ChIP assay for DRE-3 and the RelBAhRE regions of the mIL-22 promoter. **(A)** TCDD and LPS stimulate the recruitment of AhR to the DRE-3 site and **(B)** the RelBAhRE site of the mIL-22 promoter. BMM from three mice were stimulated with TCDD in the presence or absence of LPS for 6 h, and anti-AhR or anti-RelB Abs were used for immunoprecipitation. Data are expressed as the ratio of the enrichment of the promoter region target in cells treated with TCDD in combination with LPS compared with cells treated with TCDD or LPS alone. The fold enrichment of AhR and RelB was calculated relative to that of anti-IgG, serving as a negative control. qPCR was performed to analyze the levels of AhR and RelB enriched on **(A)** DRE-3 and **(B)** RelBAhRE binding sites of the IL-22 promoter. The error bars represent standard deviations from the mean of at least three experimental replicates. ^*a*^significantly higher than control.

Chromatin immunoprecipitation (ChIP) assays with BMM from WT mice were performed to study the recruitment of AhR and RelB proteins to the IL-22 promoter. The ChIP samples were analyzed by real-time qPCR and the relative enrichment levels are shown in Figure 7. The DNA binding activity of AhR to the DRE-3 site at −1,082 bp of the IL-22 promoter was elevated by TCDD, which was enhanced by the combinatorial treatment of TCDD with LPS (Figure 7A). LPS stimulated the recruitment of RelB whereas TCDD increased the occupancy of the RelBAhRE promoter region by AhR (Figure 7B). The occupancy of AhR and RelB at the RelBAhRE promoter region was enhanced by the co-treatment with LPS plus TCDD compared to LPS or TCDD alone.

## Discussion

Numerous studies demonstrated the critical role of AhR as a transcription factor regulating immune responses and cytokine gene expression (Kerkvliet, 2002). IL-22 is a member of the IL-10 family of cytokines and can be produced by both innate and adaptive immune cells (Wei et al., 2020). Here we show that BMM express and produce IL-22 after TLR stimulation, which was synergistically enhanced after ligand-dependent activation of AhR. The additional activation of AhR triggered a significant and prolonged induction of IL-22 in TLR-activated BMM. Results indicate clearly that activation of AhR signaling can change the regular TLR- and NF-κB-mediated response supporting recent findings in AhR^−/−^ mice and LPS-activated DCs (Wu et al., 2011; Kado et al., 2017). As a consequence, the usually transient effect of LPS on IL-22 can be significantly increased and prolonged by the simultaneous activation of AhR. Noteworthy, the TLR3 ligand poly(I:C) had no effect on IL-22 expression and did not synergize the effect of TCDD in BMM. Unlike other TLRs, TLR3 signals solely through TIR-domain-containing adapter-inducing interferon-β (TRIF) and not *via* Myeloid differentiation primary response 88 (MyD88) (Kawasaki and Kawai, 2014). The result implies that the synergistic effects of AhR and TLR ligands are limited to TLR ligands which signal through MyD88. Our previous study in human DC, however, showed that poly(I:C) may induce and synergize with AhR signaling to activate the expression of IL-1ß and CCL1 (Kado et al., 2017).

**FIGURE 8:**
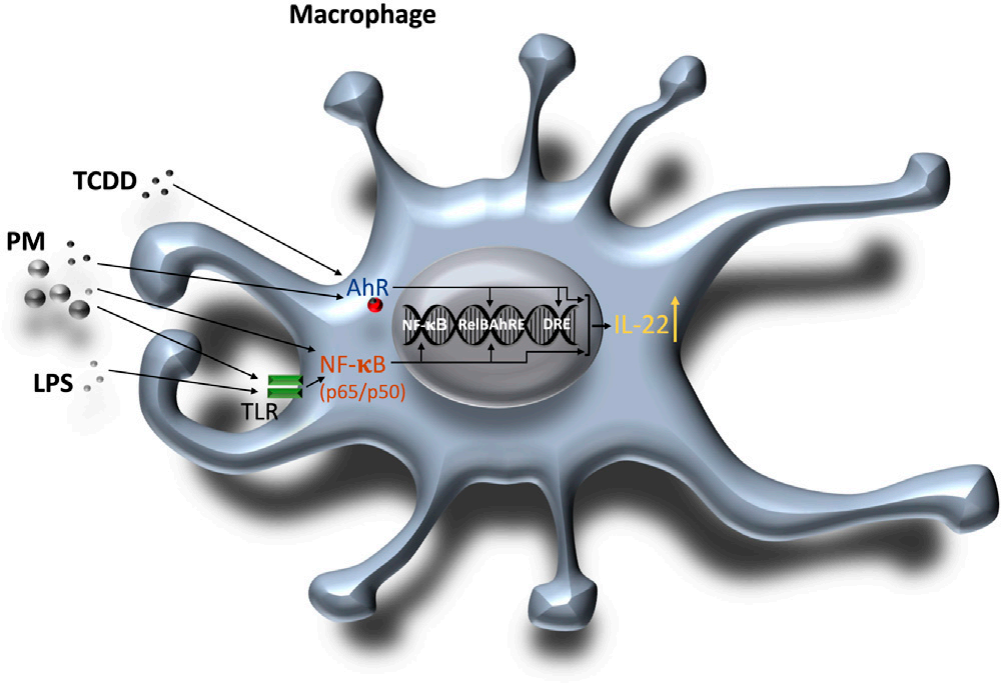
Schematic illustration of the combinatorial regulation of mouse IL-22 *via* AhR and TLR/NF-κB in macrophages. 1) AhR ligands such as TCDD or PM containing PAHs activate the AhR signaling pathway and enhance DNA binding of AhR and RelB on the IL-22 promoter. PM derived from various sources like traffic emissions, combustion or wildfires may also contain components such as endotoxin or metals which activate TLR and NF-κB. 2) TLR ligands including LPS bind to TLR and activate NF-κB (p65/p50) inducing DNA binding activity of NF-κB and RelB. Activation of both AhR and NF-κB signaling leads to a synergistic induction of IL-22.

AhR has been demonstrated to affect Th17 cell differentiation and to mediate the production of IL-17A and IL-22 in Th17 cells (Quintana et al., 2008; Veldhoen et al., 2009). The production of IL-22 by CD4^+^ T cells was dependent on the interaction AhR with RORγt which may facilitate the binding of AhR and activate the IL-22 promoter (Yeste et al., 2014). In the current study inhibitor studies and analysis of BMM derived from knockout mice confirmed that the induction of IL-22 by ligands of the AhR and TLR depend on the expression of AhR, but was not affected by the inhibition of RORγt or STAT3. The results indicate that induction of IL-22 expression in BMM via AhR is independent from RORyt and STAT3. In contrast, the TCDD-induced expression of IL-22 in IL-21-stimulated CD4^+^ T cells required RORγt and STAT3 as reported earlier (Kiss et al., 2011; Yeste et al., 2014), indicating a different mechanism of IL-22 regulation between myeloid derived macrophages and lymphoid T cells. Quintana’s group identified 3 putative binding sites for AhR upstream of the human IL-22 promoter (Yeste et al., 2014). This agrees with the identification of 3 consensus DRE sequences within 3,455 bp upstream of the start site of the mouse *Il-22* gene. The DRE sequence closest to the start site as well as a RelBAhRE DNA binding site were necessary to mediate the synergistic effects of LPS and TCDD to activate the IL-22 promoter in BMM. The mutation of the NF-κB element caused a reduced LPS-induced IL-22 promoter activity, but did not affect the synergistic effect with TCDD. However, gene silencing of RelA suppressed the TCDD- and LPS-induced mRNA expression of IL-22 in BMM. Noteworthy, RelA has been found to regulate the transcriptional activity of RelB and may also control the expression of AhR (Vogel et al., 2014; Bren et al., 2001), which may ultimately influence the effects of ligands activating TLR or AhR signaling. Results from ChIP assays indicated the enhanced recruitment of AhR and RelB to the IL-22 promoter stimulated by TCDD and LPS which supports the interaction of AhR and NF-κB RelB. A hypothetical scheme of the combinatorial regulation of IL-22 through AhR and NF-κB signaling pathways is illustrated in Figure 8. It is not yet clear whether it is the interaction of the two proteins or chromatin remodeling of the locus by NF-κB RelB that actually facilitates the binding of AhR and enables the synergistic effects of TLR and AhR ligands on IL-22 expression. The results of the current study also showed that exposure to PM derived from TRAP and wildfire smoke significantly induce the expression of IL-22. This is in line with a recent study reporting an increased production of IL-22 in peripheral blood mononuclear cells (PBMCs) from nonallergic non-asthmatic and allergic asthmatic patients after treatment with diesel exhaust particle (DEP)-PAH and Benzo[a]pyrene (B[a]P) (Plé et al., 2015). Noteworthy, PAH-induced IL-22 levels in asthmatic patients were higher than in healthy subjects. In addition to AhR-dependent signaling, the authors reported an AhR-independent pathway involving Mitogen-activated protein kinase (MAPK) and Phosphoinositide 3-kinase (PI3K) in DEP- and BaP-induced activation of IL-22 in human mononuclear cells. In the current study we found that PAH containing PM from TRAP and wildfire may not only activate AhR but also activate NF-κB signaling which would explain the strong increase of IL-22 in PM-treated BMM. Whereas, the exposure to endogenous ligands like FICZ or the prototypical ligand TCDD may totally depend upon AhR, environmentally abundant AhR ligands in mixtures such as PM containing PAHs and metals may involve additional pathways including NF-κB to dysregulate the expression of cytokines like IL-22. Recently, we were able to identify components in wildfire ash samples which correlated with activation of AhR signaling and the induction of CYP1A1 and IL-8 in macrophages (Young et al., 2021). Wildfire smoke contains numerous hazardous chemicals which may lead to adverse health effects, including potential AhR ligands such as PAHs and dioxin-like chemicals (Keir et al., 2017; Beitel et al., 2020). PM collected in urban areas and from engine emission have also been found to contain significant amounts of PAHs and induce the expression of CYP1A1 and inflammatory markers such as IL-8 in an AhR-dependent manner (Vogel et al., 2019b; Yuan et al., 2020; Li et al., 2021). Emissions from wildfires may have a substantial impact on the PM_2.5_ concentration of ambient PM samples collected during wildfire events. The compositional intercomparison of the wildfire and TRAP PM samples showed significant differences in trace and crustal elements and increased levels of organic carbon in wildfire PM vs TRAP PM. More research is needed to clearly identify the chemical composition and the bioactive compounds in complex mixtures from wildfire and TRAP related sources, which are responsible to mediate human health effects. The fact that AhR may act as a sensor of environmental signals in the form of PM components suggest that the dysregulation of cytokines such as IL-22 and likely other inflammatory biomarkers initiates a critical step in the development of chronic diseases promoted by the exposure to air pollutants. In summary the results show that simultaneous activation of AhR and TLR signaling synergistically induce IL-22 and the mechanism of IL-22 induction in BMM may differ from lymphoid T cells which require RORyt. This indicates that it is important to define the molecular mechanisms by which AhR exerts its influence in different cell types. Finally, innate immune cells like macrophages that respond to AhR and TLR ligands with synergistic expression of IL-22 and the extent to which their production of IL-22 contributes to homeostasis and host defense or chronic inflammatory diseases and malignancies requires further investigation.

## Data Availability

The original contributions presented in the study are included in the article/supplementary files, further inquiries can be directed to the corresponding author.

## References

[B1] Andreae M. O. Merlet P. (2001). Emission of Trace Gases and Aerosols from Biomass Burning. Glob. Biogeochem. Cycles 15 (4), 955–966. 10.1029/2000gb001382

[B2] Arshad T. Mansur F. Palek R. Manzoor S. Liska V. (2020). A Double Edged Sword Role of Interleukin-22 in Wound Healing and Tissue Regeneration. Front. Immunol. 11, 2148. 10.3389/fimmu.2020.02148 33042126 PMC7527413

[B3] Banerjee K. Resat H. (2016). Constitutive Activation of STAT3 in Breast Cancer Cells: a Review. Int. J. Cancer 138, 2570–2578. 10.1002/ijc.29923 26559373 PMC4801660

[B4] Bankoti J. Rase B. Simones T. Shepherd D. M. (2010). Functional and Phenotypic Effects of AhR Activation in Inflammatory Dendritic Cells. Toxicol. Appl. Pharmacol. 246 (1-2), 18–28. 10.1016/j.taap.2010.03.013 20350561 PMC2885531

[B5] Bein K. J. Wexler A. S. (2014). A High-Efficiency, Low-Bias Method for Extracting Particulate Matter from Filter and Impactor Substrates. Atmos. Environ. 90, 87–95. 10.1016/j.atmosenv.2014.03.042

[B6] Bein K. J. Wexler A. S. (2015). Compositional Variance in Extracted Particulate Matter Using Different Filter Extraction Techniques. Atmos. Environ. 107, 24–34. 10.1016/j.atmosenv.2015.02.026

[B7] Beitel S. C. Flahr L. M. Hoppe-Jones C. Burgess J. L. Littau S. R. Gulotta J. (2020). Assessment of the Toxicity of Firefighter Exposures Using the PAH CALUX Bioassay. Environ. Int. 135, 105207. 10.1016/j.envint.2019.105207 31812113

[B8] Berg E. L. Pedersen L. R. Pride M. C. Petkova S. P. Patten K. T. Valenzuela A. E. (2020). Developmental Exposure to Near Roadway Pollution Produces Behavioral Phenotypes Relevant to Neurodevelopmental Disorders in Juvenile Rats. Transl. Psychiatry 10 (1), 289. 10.1038/s41398-020-00978-0 32807767 PMC7431542

[B9] Bren G. D. Solan N. J. Miyoshi H. Pennington K. N. Pobst L. J. Paya C. V. (2001). Transcription of the RelB Gene Is Regulated by NF-κB. Oncogene 20 (53), 7722–7733. 10.1038/sj.onc.1204868 11753650

[B10] Castañeda A. R. Pinkerton K. E. Bein K. J. Magaña-Méndez A. Yang H. T. Ashwood P. (2018). Ambient Particulate Matter Activates the Aryl Hydrocarbon Receptor in Dendritic Cells and Enhances Th17 Polarization. Toxicol. Lett. 292, 85–96. 10.1016/j.toxlet.2018.04.020 29689377 PMC5971007

[B11] Chellan B. Yan L. Sontag T. J. Reardon C. A. Hofmann Bowman M. A. (2014). IL-22 Is Induced by S100/calgranulin and Impairs Cholesterol Efflux in Macrophages by Downregulating ABCG1. J. Lipid Res. 55 (3), 443–454. 10.1194/jlr.m044305 24367046 PMC3934729

[B12] Dahlem C. Kado S. Y. He Y. Bein K. Wu D. Haarmann-Stemmann T. (2020). AHR Signaling Interacting with Nutritional Factors Regulating the Expression of Markers in Vascular Inflammation and Atherogenesis. Ijms 21 (21), 8287. 10.3390/ijms21218287 PMC7663825 33167400

[B13] Denison M. S. Soshilov A. A. He G. DeGroot D. E. Zhao B. (2011). Exactly the Same but Different: Promiscuity and Diversity in the Molecular Mechanisms of Action of the Aryl Hydrocarbon (Dioxin) Receptor. Toxicol. Sci. 124 (1), 1–22. 10.1093/toxsci/kfr218 21908767 PMC3196658

[B14] D'Evelyn S. M. Vogel C. F. A. Bein K. J. Lara B. Laing E. A. Abarca R. A. (2021). Differential Inflammatory Potential of Particulate Matter (PM) Size Fractions from Imperial Valley, CA. Atmos. Environ. 244, 117992. 10.1016/j.atmosenv.2020.117992 PMC7654835 33184556

[B15] Di Lullo G. Marcatti M. Heltai S. Brunetto E. Tresoldi C. Bondanza A. (2015). Th22 Cells Increase in Poor Prognosis Multiple Myeloma and Promote Tumor Cell Growth and Survival. Oncoimmunology 4, e1005460. 10.1080/2162402x.2015.1005460 26155400 PMC4485827

[B16] Dmitrieva-Posocco O. Dzutsev A. Posocco D. F. Hou V. Yuan W. Thovarai V. (2019). Cell-Type-Specific Responses to Interleukin-1 Control Microbial Invasion and Tumor-Elicited Inflammation in Colorectal Cancer. Immunity 50, 166–180.e7. 10.1016/j.immuni.2018.11.015 30650375 PMC6490968

[B17] Domínguez-Acosta O. Vega L. Estrada-Muñiz E. Rodríguez M. S. Gonzalez F. J. Elizondo G. (2018). Activation of Aryl Hydrocarbon Receptor Regulates the LPS/IFNγ-induced Inflammatory Response by Inducing Ubiquitin-Proteosomal and Lysosomal Degradation of RelA/p65. Biochem. Pharmacol. 155, 141–149. 10.1016/j.bcp.2018.06.016 29935959 PMC6594173

[B18] Edwards S. Zhao G. Tran J. Patten K. T. Valenzuela A. Wallis C. (2020). Pathological Cardiopulmonary Evaluation of Rats Chronically Exposed to Traffic-Related Air Pollution. Environ. Health Perspect. 128 (12), 127003. 10.1289/ehp7045 33275451 PMC7717845

[B19] Fumagalli S. Torri A. Papagna A. Citterio S. Mainoldi F. Foti M. (2016). IL-22 Is Rapidly Induced by Pathogen Recognition Receptors Stimulation in Bone-Marrow-Derived Dendritic Cells in the Absence of IL-23. Sci. Rep. 6, 33900. 10.1038/srep33900 27652524 PMC5031995

[B20] Geboes L. Dumoutier L. Kelchtermans H. Schurgers E. Mitera T. Renauld J.-C. (2009). Proinflammatory Role of the Th17 Cytokine Interleukin-22 in Collagen-Induced Arthritis in C57BL/6 Mice. Arthritis Rheum. 60 (2), 390–395. 10.1002/art.24220 19180498

[B21] Gutiérrez-Vázquez C. Quintana F. J. (2018). Regulation of the Immune Response by the Aryl Hydrocarbon Receptor. Immunity 48 (1), 19–33. 10.1016/j.immuni.2017.12.012 29343438 PMC5777317

[B22] Hansson M. Silverpil E. Lindén A. Glader P. (2013). Interleukin-22 Produced by Alveolar Macrophages during Activation of the Innate Immune Response. Inflamm. Res. 62 (6), 561–569. Epub 2013 Mar 9. PMID: 23474919. 10.1007/s00011-013-0608-1 23474919

[B23] Heinemeyer T. Wingender E. Reuter I. Hermjakob H. Kel A. E. Kel O. V. (1998). Databases on Transcriptional Regulation: TRANSFAC, TRRD and COMPEL. Nucleic Acids Res. 26, 362–367. 10.1093/nar/26.1.362 9399875 PMC147251

[B24] Hernandez P. Gronke K. Diefenbach A. (2018). A Catch-22: Interleukin-22 and Cancer. Eur. J. Immunol. 48 (1), 15–31. PMID: 29178520. 10.1002/eji.201747183 29178520

[B25] Hoffmann A. Levchenko A. Scott M. L. Baltimore D. (2002). The IκB-NF-κB Signaling Module: Temporal Control and Selective Gene Activation. Science 298 (5596), 1241–1245. 10.1126/science.1071914 12424381

[B26] Huber S. Gagliani N. Zenewicz L. A. Huber F. J. Bosurgi L. Hu B. (2012). IL-22BP Is Regulated by the Inflammasome and Modulates Tumorigenesis in the Intestine. Nature 491 (7423), 259–263. 10.1038/nature11535 23075849 PMC3493690

[B27] Ikeuchi H. Kuroiwa T. Hiramatsu N. Kaneko Y. Hiromura K. Ueki K. (2005). Expression of Interleukin-22 in Rheumatoid Arthritis: Potential Role as a Proinflammatory Cytokine. Arthritis Rheum. 52, 1037–1046. 10.1002/art.20965 15818686

[B28] Irshad S. Flores-Borja F. Lawler K. Monypenny J. Evans R. Male V. (2017). RORγt+ Innate Lymphoid Cells Promote Lymph Node Metastasis of Breast Cancers. Cancer Res. 77, 1083–1096. 10.1158/0008-5472.can-16-0598 28082403

[B29] Ishihara Y. Kado S. Y. Hoeper C. Harel S. Vogel C. F. A. (2019). Role of NF-kB RelB in Aryl Hydrocarbon Receptor-Mediated Ligand Specific Effects. Ijms 20 (11), 2652. 10.3390/ijms20112652 PMC6600526 31151139

[B30] Ishihara Y. Haarmann-Stemmann T. Kado N. Y. Vogel C. F. A. (2019). Interleukin 33 Expression Induced by Aryl Hydrocarbon Receptor in Macrophages. Toxicol. Sci. 170 (2), 404–414. 10.1093/toxsci/kfz114 31093659 PMC6657576

[B31] Iu M. Zago M. Rico de Souza A. Bouttier M. Pareek S. White J. H. (2017). RelB Attenuates Cigarette Smoke Extract-Induced Apoptosis in Association with Transcriptional Regulation of the Aryl Hydrocarbon Receptor. Free Radic. Biol. Med. 108, 19–31. 10.1016/j.freeradbiomed.2017.02.045 28254546

[B32] Jin G.-B. Moore A. J. Head J. L. Neumiller J. J. Lawrence B. P. (2010). Aryl Hydrocarbon Receptor Activation Reduces Dendritic Cell Function during Influenza Virus Infection. Toxicol. Scipmcid 116 (2), 514–522. 10.1093/toxsci/kfq153 PMC2905408 20498003

[B33] Kado S. Chang W. L. W. Chi A. N. Wolny M. Shepherd D. M. Vogel C. F. A. (2017). Aryl Hydrocarbon Receptor Signaling Modifies Toll-like Receptor-Regulated Responses in Human Dendritic Cells. Arch. Toxicol. 91 (5), 2209–2221. Erratum in: Arch Toxicol. 2017 Jul;91(7):2713. 10.1007/s00204-016-1880-y 27783115 PMC5400689

[B34] Kawasaki T. Kawai T. (2014). Toll-like Receptor Signaling Pathways. Front. Immunol. 5, 461. 10.3389/fimmu.2014.00461 25309543 PMC4174766

[B35] Ke S. Rabson A. B. Germino J. F. Gallo M. A. Tian Y. (2001). Mechanism of Suppression of Cytochrome P-450 1A1 Expression by Tumor Necrosis Factor-α and Lipopolysaccharide. J. Biol. Chem. 276 (43), 39638–39644. 10.1074/jbc.m106286200 11470802

[B36] Keir J. L. A. Akhtar U. S. Matschke D. M. J. Kirkham T. L. Chan H. M. Ayotte P. (2017). Elevated Exposures to Polycyclic Aromatic Hydrocarbons and Other Organic Mutagens in Ottawa Firefighters Participating in Emergency, On-Shift Fire Suppression. Environ. Sci. Technol. 51 (21), 12745–12755. 10.1021/acs.est.7b02850 29043785

[B37] Keir M. Yi Y. Lu T. Ghilardi N. (2020). The Role of IL-22 in Intestinal Health and Disease. J. Exp. Med. 217 (3), e20192195. 10.1084/jem.20192195 32997932 PMC7062536

[B38] Kerkvliet N. I. (2002). Recent Advances in Understanding the Mechanisms of TCDD Immunotoxicity. Int. Immunopharmacol. 2, 277–291. 10.1016/s1567-5769(01)00179-5 11811931

[B39] Kim D. W. Gazourian L. Quadri S. A. Raphaëlle R. Sherr D. H. Sonenshein G. E. (2000). The RelA NF-κB Subunit and the Aryl Hydrocarbon Receptor (AhR) Cooperate to Transactivate the C-Myc Promoter in Mammary Cells. Oncogene 19 (48), 5498–5506. 10.1038/sj.onc.1203945 11114727

[B40] Kim K. Kim G. Kim J.-Y. Yun H. J. Lim S.-C. Choi H. S. (2014). Interleukin-22 Promotes Epithelial Cell Transformation and Breast Tumorigenesis via MAP3K8 Activation. Carcinogenesis 35 (6), 1352–1361. 10.1093/carcin/bgu044 24517997

[B41] Kimura A. Naka T. Nakahama T. Chinen I. Masuda K. Nohara K. (2009). Aryl Hydrocarbon Receptor in Combination with Stat1 Regulates LPS-Induced Inflammatory Responses. J. Exp. Med. 206 (9), 2027–2035. 10.1084/jem.20090560 19703987 PMC2737163

[B42] Kirchberger S. Royston D. J. Boulard O. Thornton E. Franchini F. Szabady R. L. (2013). Innate Lymphoid Cells Sustain colon Cancer through Production of Interleukin-22 in a Mouse Model. J. Exp. Med. 210, 917–931. 10.1084/jem.20122308 23589566 PMC3646494

[B43] Kiss E. A. Vonarbourg C. Kopfmann S. Hobeika E. Finke D. Esser C. (2011). Natural Aryl Hydrocarbon Receptor Ligands Control Organogenesis of Intestinal Lymphoid Follicles. Science 334 (6062), 1561–1565. 10.1126/science.1214914 22033518

[B44] Lahoti T. S. Boyer J. A. Kusnadi A. Muku G. E. Murray I. A. Perdew G. H. (2015). Aryl Hydrocarbon Receptor Activation Synergistically Induces Lipopolysaccharide-Mediated Expression of Proinflammatory Chemokine (C-c Motif) Ligand 20. Toxicol. Sci. 148 (1), 229–240. 10.1093/toxsci/kfv178 26259605 PMC4731409

[B45] Lee J. S. Cella M. McDonald K. G. Garlanda C. Kennedy G. D. Nukaya M. (2011). AHR Drives the Development of Gut ILC22 Cells and Postnatal Lymphoid Tissues via Pathways Dependent on and Independent of Notch. Nat. Immunol. 13 (2), 144–151. 10.1038/ni.2187 22101730 PMC3468413

[B46] Lemieux P. M. Lutes C. C. Santoianni D. A. (2004). Emissions of Organic Air Toxics from Open Burning: a Comprehensive Review. Prog. Energ. Combustion Sci. 30 (1), 1–32. 10.1016/j.pecs.2003.08.001

[B47] Li Y. Xue J. Peppers J. Kado N. Y. Vogel C. F. A. Alaimo C. P. (2021). Chemical and Toxicological Properties of Emissions from a Light-Duty Compressed Natural Gas Vehicle Fueled with Renewable Natural Gas. Environ. Sci. Technol. 55 (5), 2820–2830. 10.1021/acs.est.0c04962 33555876 PMC8284984

[B48] Lim C. Savan R. (2014). The Role of the IL-22/IL-22R1 axis in Cancer. Cytokine Growth Factor. Rev. 25 (3), 257–271. 10.1016/j.cytogfr.2014.04.005 24856143

[B49] Ma H. L. Liang S. Li J. Napierata L. Brown T. Benoit S. (2008). IL-22 Is Required for Th17 Cell-Mediated Pathology in a Mouse Model of Psoriasis-like Skin Inflammation. J. Clin. Invest. 118 (2), 597–607. 10.1172/JCI33263 18202747 PMC2200300

[B50] Markota A. Endres S. Kobold S. (2018). Targeting Interleukin-22 for Cancer Therapy. Hum. Vaccin. Immunother. 14 (8), 2012–2015. 10.1080/21645515.2018.1461300 29617184 PMC6149728

[B51] Marshall N. B. Kerkvliet N. I. (2010). Dioxin and Immune Regulation. Ann. N. Y Acad. Sci. 1183, 25–37. 10.1111/j.1749-6632.2009.05125.x 20146706 PMC4263666

[B52] Mashiko S. Bouguermouh S. Rubio M. Baba N. Bissonnette R. Sarfati M. (2015). Human Mast Cells Are Major IL-22 Producers in Patients with Psoriasis and Atopic Dermatitis. J. Allergy Clin. Immunol. 136, 351–359. e351. 10.1016/j.jaci.2015.01.033 25792465

[B53] Matthews J. Gustafsson J. A. (2006). Estrogen Receptor and Aryl Hydrocarbon Receptor Signaling Pathways. Nucl. Recept Signal. 4, e016. 10.1621/nrs.04016 16862222 PMC1513070

[B54] Mogensen T. H. (2009). Pathogen Recognition and Inflammatory Signaling in Innate Immune Defenses. Clin. Microbiol. Rev. 22 (2), 240–273. 10.1128/cmr.00046-08 19366914 PMC2668232

[B55] O'Driscoll C. A. Gallo M. E. Hoffmann E. J. Fechner J. H. Schauer J. J. Bradfield C. A. (2018). Polycyclic Aromatic Hydrocarbons (PAHs) Present in Ambient Urban Dust Drive Proinflammatory T Cell and Dendritic Cell Responses via the Aryl Hydrocarbon Receptor (AHR) *In Vitro* . PLoS One 13 (12), e0209690. 10.1371/journal.pone.0209690 30576387 PMC6303068

[B56] Oesch-Bartlomowicz B. Huelster A. Wiss O. Antoniou-Lipfert P. Dietrich C. Arand M. (2005). Aryl Hydrocarbon Receptor Activation by cAMP vs. Dioxin: Divergent Signaling Pathways. Proc. Natl. Acad. Sci. 102 (26), 9218–9223. 10.1073/pnas.0503488102 15972329 PMC1154791

[B57] Øvrevik J. Låg M. Lecureur V. Gilot D. Lagadic-Gossmann D. Refsnes M. (2014). AhR and Arnt Differentially Regulate NF-κB Signaling and Chemokine Responses in Human Bronchial Epithelial Cells. Cel. Commun. Signal 12, 48. 10.1186/s12964-014-0048-8 PMC4222560 25201625

[B58] Palm B. B. Peng Q. Fredrickson C. D. Lee B. H. Garofalo L. A. Pothier M. A. (2020). Quantification of Organic Aerosol and Brown Carbon Evolution in Fresh Wildfire Plumes. Proc. Natl. Acad. Sci. USA 117 (47), 29469–29477. 10.1073/pnas.2012218117 33148807 PMC7703578

[B59] Patten K. T. González E. A. Valenzuela A. Berg E. Wallis C. Garbow J. R. (2020). Effects of Early Life Exposure to Traffic-Related Air Pollution on Brain Development in Juvenile Sprague-Dawley Rats. Transl. Psychiatry 10, 166. 10.1038/s41398-020-0845-3 32483143 PMC7264203

[B60] Patten K. T. Valenzuela A. E. Wallis C. Berg E. L. Silverman J. L. Bein K. J. (2021). The Effects of Chronic Exposure to Ambient Traffic-Related Air Pollution on Alzheimer's Disease Phenotypes in Wildtype and Genetically Predisposed Male and Female Rats. Environ. Health Perspect. 129 (5), 57005. 10.1289/EHP8905 33971107 PMC8110309

[B61] Perusina Lanfranca M. Lin Y. Fang J. Zou W. Frankel T. (2016). Biological and Pathological Activities of Interleukin-22. J. Mol. Med. 94 (5), 523–534. 10.1007/s00109-016-1391-6 26923718 PMC4860114

[B62] Plé C. Fan Y. Ait Yahia S. Vorng H. Everaere L. Chenivesse C. (2015). Polycyclic Aromatic Hydrocarbons Reciprocally Regulate IL-22 and IL-17 Cytokines in Peripheral Blood Mononuclear Cells from Both Healthy and Asthmatic Subjects. PLoS One 10 (4), e0122372. 10.1371/journal.pone.0122372 25860963 PMC4393221

[B63] Puga A. Barnes S. J. Chang C.-y. Zhu H. Nephew K. P. Khan S. A. (2000). Activation of Transcription Factors Activator Protein-1 and Nuclear Factor-κB by 2,3,7,8-Tetrachlorodibenzo-P-Dioxin. Biochem. Pharmacol. 59 (8), 997–1005. 10.1016/s0006-2952(99)00406-2 10692565

[B64] Qiu J. Heller J. J. Guo X. Chen Z.-m. E. Fish K. Fu Y.-X. (2012). The Aryl Hydrocarbon Receptor Regulates Gut Immunity through Modulation of Innate Lymphoid Cells. Immunity 36 (1), 92–104. 10.1016/j.immuni.2011.11.011 22177117 PMC3268875

[B65] Quintana F. J. Basso A. S. Iglesias A. H. Korn T. Farez M. F. Bettelli E. (2008). Control of Treg and TH17 Cell Differentiation by the Aryl Hydrocarbon Receptor. Nature 453 (7191), 65–71. 10.1038/nature06880 18362915

[B66] Rattik S. Hultman K. Rauch U. Söderberg I. Sundius L. Ljungcrantz I. (2015). IL-22 Affects Smooth Muscle Cell Phenotype and Plaque Formation in Apolipoprotein E Knockout Mice. Atherosclerosis 242 (2), 506–514. Epub 2015 Aug 11. PMID: 26298743. 10.1016/j.atherosclerosis.2015.08.006 26298743

[B67] Rothhammer V. Quintana F. J. (2019). The Aryl Hydrocarbon Receptor: an Environmental Sensor Integrating Immune Responses in Health and Disease. Nat. Rev. Immunol. 19 (3), 184–197. 10.1038/s41577-019-0125-8 30718831

[B68] Salisbury R. L. Sulentic C. E. (2015). The AhR and NF-κB/Rel Proteins Mediate the Inhibitory Effect of 2,3,7,8-Tetrachlorodibenzo-P-Dioxin on the 3' Immunoglobulin Heavy Chain Regulatory Region. Toxicol. Sci. 148 (2), 443–459. pii: kfv193. 10.1093/toxsci/kfv193 26377645 PMC5009439

[B69] Sonnenberg G. F. Fouser L. A. Artis D. (2011). Border Patrol: Regulation of Immunity, Inflammation and Tissue Homeostasis at Barrier Surfaces by IL-22. Nat. Immunol. 12 (5), 383–390. 10.1038/ni.2025 21502992

[B70] Stockinger B. Meglio P. D. Gialitakis M. Duarte J. H. (2014). The Aryl Hydrocarbon Receptor: Multitasking in the Immune System. Annu. Rev. Immunol. 32, 403–432. 10.1146/annurev-immunol-032713-120245 24655296

[B71] Sulentic C. E. Kang J. S. Na Y. J. Kaminski N. E. (2004). Interactions at a Dioxin Responsive Element (DRE) and an Overlapping kappaB Site within the Hs4 Domain of the 3'alpha Immunoglobulin Heavy Chain Enhancer. Toxicology 200 (2-3), 235–246. 10.1016/j.tox.2004.03.015 15212819

[B72] Tian Y. Rabson A. B. Gallo M. A. (2002). Ah Receptor and NF-kappaB Interactions: Mechanisms and Physiological Implications. Chem. Biol. Interact 141 (1-2), 97–115. 10.1016/s0009-2797(02)00068-6 12213387

[B73] Veldhoen M. Hirota K. Christensen J. O'Garra A. Stockinger B. (2009). Natural Agonists for Aryl Hydrocarbon Receptor in Culture Medium Are Essential for Optimal Differentiation of Th17 T Cells. J. Exp. Med. 206 (1), 43–49. 10.1084/jem.20081438 19114668 PMC2626686

[B74] Vogel C. F. A. Sciullo E. Li W. Wong P. Lazennec G. Matsumura F. (2007). RelB, a New Partner of Aryl Hydrocarbon Receptor-Mediated Transcription. Mol. Endocrinol. 21 (12), 2941–2955. 10.1210/me.2007-0211 17823304 PMC2346533

[B75] Vogel C. F. A. Goth S. R. Dong B. Pessah I. N. Matsumura F. (2008). Aryl Hydrocarbon Receptor Signaling Mediates Expression of Indoleamine 2,3-dioxygenase. Biochem. Biophys. Res. Commun. 375, 331–335. 10.1016/j.bbrc.2008.07.156 18694728 PMC2583959

[B76] Vogel C. F. A. Li W. Wu D. Miller J. K. Sweeney C. Lazennec G. (2011). Interaction of Aryl Hydrocarbon Receptor and NF-κB Subunit RelB in Breast Cancer Is Associated with Interleukin-8 Overexpression. Arch. Biochem. Biophys. 512 (1), 78–86. 10.1016/j.abb.2011.05.011 21640702 PMC3135412

[B77] Vogel C. F. A. Wu D. Goth S. R. Baek J. Lollies A. Domhardt R. (2013). Aryl Hydrocarbon Receptor Signaling Regulates NF‐κB RelB Activation during Dendritic‐cell Differentiation. Immunol. Cel. Biol. 91 (9), 568–575. 10.1038/icb.2013.43 PMC3806313 23999131

[B78] Vogel C. F. A. Khan E. M. Leung P. S. C. Gershwin M. E. Chang W. L. W. Wu D. (2014). Cross-talk between Aryl Hydrocarbon Receptor and the Inflammatory Response. J. Biol. Chem. 289 (3), 1866–1875. 10.1074/jbc.m113.505578 24302727 PMC3894361

[B79] Vogel C. F. A. Ishihara Y. Campbell C. E. Kado S. Y. Nguyen-Chi A. Sweeney C. (2019). A Protective Role of Aryl Hydrocarbon Receptor Repressor in Inflammation and Tumor Growth. Cancers 11 (5), 589. 10.3390/cancers11050589 PMC6563059 31035533

[B80] Vogel C. F. A. Kado S. Y. Kobayashi R. Liu X. Wong P. Na K. (2019). Inflammatory Marker and Aryl Hydrocarbon Receptor-dependent Responses in Human Macrophages Exposed to Emissions from Biodiesel Fuels. Chemosphere 220, 993–1002. 10.1016/j.chemosphere.2018.12.178 31543100 PMC6858841

[B81] Vogel C. F. A. Van Winkle L. S. Esser C. Haarmann-Stemmann T. (2020). The Aryl Hydrocarbon Receptor as a Target of Environmental Stressors - Implications for Pollution Mediated Stress and Inflammatory Responses. Redox Biol. 34, 101530. 10.1016/j.redox.2020.101530 32354640 PMC7327980

[B82] Voigt C. May P. Gottschlich A. Markota A. Wenk D. Gerlach I. (2017). Cancer Cells Induce Interleukin-22 Production from Memory CD4+ T Cells via Interleukin-1 to Promote Tumor Growth. Proc. Natl. Acad. Sci. USA 114 (49), 12994–12999. 10.1073/pnas.1705165114 29150554 PMC5724250

[B83] Wei H.-X. Wang B. Li B. (2020). IL-10 and IL-22 in Mucosal Immunity: Driving Protection and Pathology. Front. Immunol. 11, 1315. 10.3389/fimmu.2020.01315 32670290 PMC7332769

[B84] Wright E. J. Pereira De Castro K. Joshi A. D. Elferink C. J. (2017). Canonical and Non-canonical Aryl Hydrocarbon Receptor Signaling Pathways. Curr. Opin. Toxicol. 2, 87–92. PMC7158745. 10.1016/j.cotox.2017.01.001 32296737 PMC7158745

[B85] Wu D. Li W. Lok P. Matsumura F. Adam Vogel C. F. (2011). AhR Deficiency Impairs Expression of LPS-Induced Inflammatory Genes in Mice. Biochem. Biophys. Res. Commun. 410 (2), 358–363. 10.1016/j.bbrc.2011.06.018 21683686 PMC3137281

[B86] Yeste A. Mascanfroni I. D. Nadeau M. Burns E. J. Tukpah A.-M. Santiago A. (2014). IL-21 Induces IL-22 Production in CD4+ T Cells. Nat. Commun. 5, 3753. 10.1038/ncomms4753 24796415 PMC4157605

[B87] Young T. M. Black G. P. Wong L. Bloszies C. S. Fiehn O. He G. (2021). Identifying Toxicologically Significant Compounds in Urban Wildfire Ash Using *In Vitro* Bioassays and High-Resolution Mass Spectrometry. Environ. Sci. Technol. 55, 3657–3667. Epub ahead of print. PMID: 33647203. 10.1021/acs.est.0c06712 33647203 PMC8351470

[B88] Yuan W. Fulgar C. C. Sun X. Vogel C. F. A. Wu C.-W. Zhang Q. (2020). *In Vivo* and *In Vitro* Inflammatory Responses to fine Particulate Matter (PM2.5) from China and California. Toxicol. Lett. 328, 52–60. 10.1016/j.toxlet.2020.04.010 32320776 PMC7641014

[B89] Zenewicz L. A. Yancopoulos G. D. Valenzuela D. M. Murphy A. J. Stevens S. Flavell R. A. (2008). Innate and Adaptive Interleukin-22 Protects Mice from Inflammatory Bowel Disease. Immunity 29 (6), 947–957. 10.1016/j.immuni.2008.11.003 19100701 PMC3269819

[B90] Zhang Y. Liu C. Gao J. Shao S. Cui Y. Yin S. (2020). IL-22 Promotes Tumor Growth of Breast Cancer Cells in Mice. Aging 12 (13), 13354–13364. 10.18632/aging.103439 32649314 PMC7377855

[B91] Zhao J. Liu H. Zhang X. Zhang W. Liu L. Yu Y. (2021). Tumor Cells Interleukin-22 Expression Associates with Elevated Tumor-Associated Macrophages Infiltrating and Poor Prognosis in Patients with Breast Cancer. Cancer Biother. Radiopharm. 36 (2), 160–166. 10.1089/cbr.2020.3794 33090014

[B92] Zhu J. Luo L. Tian L. Yin S. Ma X. Cheng S. (2018). Aryl Hydrocarbon Receptor Promotes IL-10 Expression in Inflammatory Macrophages through Src-STAT3 Signaling Pathway. Front. Immunol. 9, 2033. 10.3389/fimmu.2018.02033 30283437 PMC6156150

[B93] Zindl C. L. Lai J.-F. Lee Y. K. Maynard C. L. Harbour S. N. Ouyang W. (2013). IL-22-producing Neutrophils Contribute to Antimicrobial Defense and Restitution of Colonic Epithelial Integrity during Colitis. Proc. Natl. Acad. Sci. 110 (31), 12768–12773. 10.1073/pnas.1300318110 23781104 PMC3732935

